# Cerebrovascular reactivity measurements using simultaneous ^15^O-water PET and ASL MRI: Impacts of arterial transit time, labeling efficiency, and hematocrit^☆^

**DOI:** 10.1016/j.neuroimage.2021.117955

**Published:** 2021-03-11

**Authors:** Moss Y Zhao, Audrey P Fan, David Yen-Ting Chen, Magdalena J. Sokolska, Jia Guo, Yosuke Ishii, David D Shin, Mohammad Mehdi Khalighi, Dawn Holley, Kim Halbert, Andrea Otte, Brittney Williams, Taghi Rostami, Jun-Hyung Park, Bin Shen, Greg Zaharchuk

**Affiliations:** a Department of Radiology, Stanford University, Stanford, CA, United States; b Department of Biomedical Engineering, University of California Davis, Davis, CA, USA; c Department of Neurology, University of California Davis, Davis, CA, USA; d Department of Medical Imaging, Taipei Medical University – Shuan-Ho Hospital, New Taipei City, Taiwan; e Department of Radiology, School of Medicine, College of Medicine, Taipei Medical University, Taipei, Taiwan; f Medical Physics and Biomedical Engineering, University College London Hospitals, London, United Kingdom; g Department of Bioengineering, University of California Riverside, Riverside, CA, United States; h Department of Neurosurgery, Tokyo Medical and Dental University, Tokyo, Japan; i GE Healthcare, Menlo Park, CA, United States; j Department of Bioengineering, Stanford University, Stanford, CA, United States

**Keywords:** Cerebrovascular reactivity, Cerebrovascular reserve, Cerebral blood flow, Positron emission tomography, Magnetic resonance imaging, Pseudo-continuous arterial spin labeling, Velocity selective arterial spin labeling, PET/MRI

## Abstract

Cerebrovascular reactivity (CVR) reflects the capacity of the brain to meet changing physiological demands and can predict the risk of cerebrovascular diseases. CVR can be obtained by measuring the change in cerebral blood flow (CBF) during a brain stress test where CBF is altered by a vasodilator such as acetazolamide. Although the gold standard to quantify CBF is PET imaging, the procedure is invasive and inaccessible to most patients. Arterial spin labeling (ASL) is a non-invasive and quantitative MRI method to measure CBF, and a consensus guideline has been published for the clinical application of ASL. Despite single post labeling delay (PLD) pseudo-continuous ASL (PCASL) being the recommended ASL technique for CBF quantification, it is sensitive to variations to the arterial transit time (ATT) and labeling efficiency induced by the vasodilator in CVR studies. Multi-PLD ASL controls for the changes in ATT, and velocity selective ASL is in theory insensitive to both ATT and labeling efficiency. Here we investigate CVR using simultaneous ^15^O-water PET and ASL MRI data from 19 healthy subjects. CVR and CBF measured by the ASL techniques were compared using PET as the reference technique. The impacts of blood T1 and labeling efficiency on ASL were assessed using individual measurements of hematocrit and flow velocity data of the carotid and vertebral arteries measured using phase-contrast MRI. We found that multi-PLD PCASL is the ASL technique most consistent with PET for CVR quantification (group mean CVR of the whole brain = 42 ± 19% and 40 ± 18% respectively). Single-PLD ASL underestimated the CVR of the whole brain significantly by 15 ± 10% compared with PET (*p*<0.01, paired t-test). Changes in ATT pre- and post-acetazolamide was the principal factor affecting ASL-based CVR quantification. Variations in labeling efficiency and blood T1 had negligible effects.

## 1. Introduction

Cerebrovascular reactivity (CVR) is an important biomarker that can predict the risk of cerebrovascular diseases (Markus and Cullinane, 2001). CVR reflects the ability of cerebral blood flow (CBF) to change when stressed and is defined as the relative change in CBF following a vasoactive stimulus (Fierstra et al., 2013). The terms cerebrovascular reactivity and cerebrovascular reserve are frequently used to describe the CBF change in response to vasodilation or vasoconstriction. According to the definition, cerebrovascular reactivity refers to the process of changing CBF induced by a stimulus and is the focus of this work; cerebrovascular reserve refers to the maximum capacity for CBF to reach. To determine CVR, CBF is measured before and after the vasoactive stimulus using medical imaging modalities such as transcranial Doppler (TCD) ultrasound, positron emission tomography (PET) imaging, and magnetic resonance imaging (MRI) (Endo et al., 2006; Pinto et al., 2016; Kassner et al., 2010; Totaro et al., 1999). While breath-hold TCD is by far the most commonly used clinical technique for CVR measurements, it is limited by the inability to determine the local CVR in specific brain regions (Gupta et al., 2012). PET has been considered the gold standard technique for CBF quantification due to its high accuracy and reproducibility (Fan et al., 2016). Since two CBF measurements (before and after the vasoactive stimulus) are needed for the quantification of CVR, ^15^O-water is the preferred radiotracer for PET-based CVR measurement due to its short half-life (2.04 min), allowing separate CBF images to be obtained before and after the administration of the vasodilator. However, the tracer’s short half-life also limits the widespread application of PET-based CVR measurements in most patients, since it requires considerable infrastructure and co-localization of scanner and cyclotron.

MRI is a non-ionizing modality that is more widely available than PET in modern clinics and can measure CBF using non-contrast arterial spin labeling (ASL) techniques (Detre et al., 1992). A consensus paper on ASL recommended single post-label delay pseudo-continuous ASL (single-PLD PCASL) for clinical applications, though it is not clear that this method is optimal for the quantification of CVR (Alsop et al., 2015). In most ASL techniques, radiofrequency pulses are applied in a plane proximal to the brain to create magnetically labeled blood water as an endogenous tracer for CBF quantification (Haller et al., 2016). While single-PLD PCASL has achieved high accuracy and reproducibility in resting-state CBF measurements (Mutsaerts et al., 2014), its application for CVR measurement may be more challenging due to changes in arterial transit time (ATT) and inversion efficiency after the administration of the vasodilator (Maccotta et al., 1997), (Aslan et al., 2010). In a recent review paper, the authors argued that neglecting the changes in the ATT and the inversion efficiency of the single-PLD PCASL technique would result in the underestimation of CVR (Liu et al., 2018), making the single-PLD PCASL a less accurate technique for ASL-based CVR measurements. ATT represents the time duration between the labeling of the blood and its deposition in the capillary bed and should be accounted for in order to obtain accurate CBF. Likewise, labeling efficiency will change with blood flow velocity at the labeling plane and could also affect quantification. The effects of ATT changes may be mitigated by using a multi-PLD ASL image acquisition whereby images at different PLDs are acquired, with the data then fitted to a general kinetic model for ATT and CBF (Cohen et al., 2020), while changes in inversion efficiency can be modeled if blood flow velocity data are available.

Velocity selective ASL (VSASL) is an advanced ASL technique that eliminates the issues of the variations in transit time and labeling efficiency (Wong et al., 2006). Since pulsed VSASL only labels the spins moving faster than a pre-defined cut-off velocity *Vc*, as opposed to the spatially selective labeling in PCASL that requires a spatial gap between the imaging and labeling sites, the resultant image is theoretically insensitive to ATT. When a specific VSASL sequence is applied to reduce the eddy current sensitivities, such as the method that developed by Guo et al., the labeling efficiency is relatively stable in the presence of B1 and B0 field inhomogeneities or blood flow velocity variations (Guo et al., 2015), (Qin and van Zijl, 2016). Given these advantages, VSASL may be well-suited for CVR quantification.

In this work, using acetazolamide (ACZ) as the vasodilator and a simultaneous PET/MRI scanner as the imaging modality, we (1) compared CVR measured by single-PLD PCASL, multi-PLD PCASL, and VSASL using ^15^O-water PET as a reference and (2) investigated the effects of ATT, T1 relaxation of arterial blood, and labeling efficiency on CVR quantification.

## 2. Methods

**Fig. 1.**
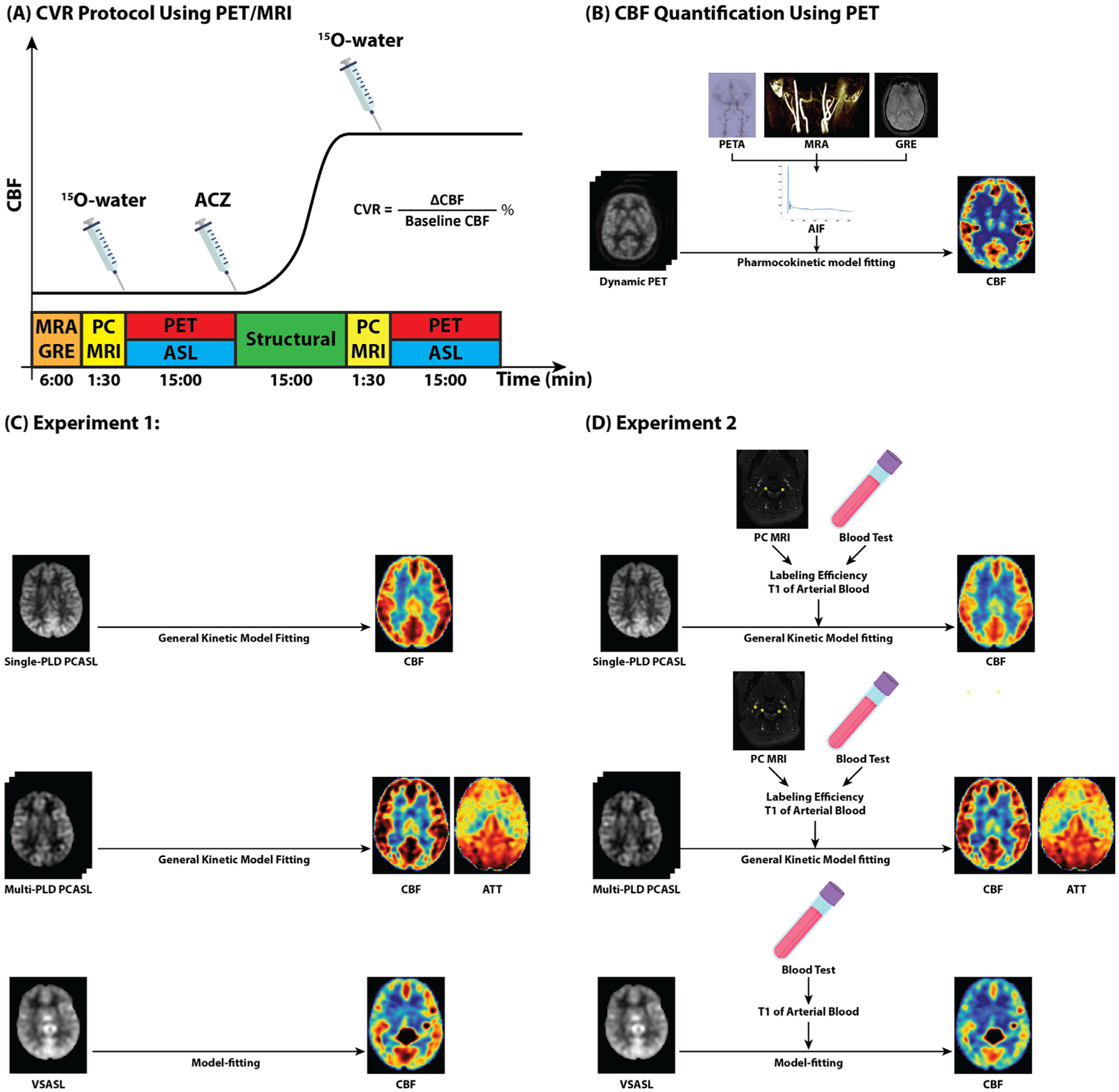
(A) Design of the PET/MRI CVR protocol. MRA and GRE were performed to identify the imaging slice of PC MRI and the labeling plane of ASL scans as well as to determine the regions of interest for the AIF calculation. PC MRI data were collected before and 15 min after ACZ administration to measure the blood flow and velocity. Simultaneous PET/ASL data were acquired for CBF and CVR quantification. T1-weighted structural scans were collected for registration. (B) PET CBF was computed by fitting a single-compartment pharmacokinetic model to the dynamic PET data. It was the used as the reference for comparison with CBF and CVR of ASL methods in both Experiment 1 and 2. PETA: PET angiogram. (C) In Experiment 1, CBF and CVR of ASL were quantified using general kinetic model fitting and assuming global T1 of blood (1.65 s) and labeling efficiency (85%) for all subjects. (D) In Experiment 2, CBF and CVR of ASL were quantified using general kinetic model fitting and estimated individual T1 of blood (based on hematocrit) and labeling efficiency values based on blood velocity measured from PC MRI data.

The study was performed in compliance with the regulations of the institutional review board of Stanford University. Simultaneous PET/MRI data were collected from 19 healthy subjects (25 – 66 years, 8 males). Exclusion criteria included kidney function impairment, pregnancy, history of brain injury, and contraindications to MRI and ACZ. The procedures were conducted according to the Declaration of Helsinki. All PET/MRI data were collected on a simultaneous time of flight (TOF) enabled 3.0T PET/MRI system (Signa, GE Healthcare, Waukesha, WI, USA) after the subjects had given written informed consent. Each subject underwent antecubital placement of a venous cannula and PET/MRI with ACZ administration. ACZ was injected during the scan at a dose of 15 mg/kg of body weight with a maximum dose of 1000 mg. All subjects were instructed to refrain from food and beverage containing caffeine six hours before the experiment (Addicott et al., 2009). Each subject received two PET/MRI scans: one at baseline (pre-ACZ) and one after the injection of ACZ (post-ACZ). Fig. 1 A shows the various imaging scans in the CVR measurement.

### 2.1. Vital signs and blood test

The blood pressure, heart rate, and hemoglobin levels were measured before the imaging session. A venous blood sample (10 ml) was drawn from each subject, and the hemoglobin and hematocrit levels were estimated using the HemoPoint® H2 Hemoglobin Analyzer (Medical Device Depot, Inc., Ellicott City, MD, USA).

### 2.2. PET experiments

Each subject received 862 ± 123 MBq of ^15^O-water before and 15 min after the administration of ACZ. PET image acquisition commenced with the tracer injection for 15 min using the parameter listed in Table 1. All PET scans covered the full brain region.

Dynamic PET frames over ten minutes (30 × 1, 10 × 3, 12 × 5, 12 × 10, 12 × 30 s) after the injection of the tracer were reconstructed using a TOF-ordered subset expectation maximization algorithm with 3 iterations and 28 subsets. Images were corrected for decay, scatter, random counts, dead time, and point spread function compensation. A 4 mm FWHM Gaussian filter was used to smooth the PET images in reconstruction. Attenuation correction was achieved using a two-point Dixon MRI acquisition and an atlas based algorithm (Ishii et al., 2019).

The reconstructed dynamic PET data were used to compute the pre- and post-ACZ voxel-wise CBF of each subject. As shown in Fig. 1 B, an image-derived arterial input function (AIF) was estimated from dynamic PET data in the carotid arteries including corrections for spill-in and out artifacts using the high-resolution segmentation of the cervical MRA and GRE data for masking (Khalighi et al., 2018). This AIF and dynamic PET data were then incorporated into the one-compartment pharmacokinetic model to quantify voxel-wise CBF (Zhou et al., 2001). The model was implemented in the spatially regularized Variational Bayesian Inference framework in FSL (Chappell et al., 2009), (Groves et al., 2009). Finally, a 2D median filter (5 × 5 voxels) was applied to the estimated CBF map to reduce the impulse noise.

### 2.3. MRI experiments

All MRI acquisition parameters are listed in Table 1. As shown in Fig. 1 A, MR angiographic (MRA) and gradient echo (GRE) scans were performed before the administration of PET tracer. The coverage of the MRA scan was a region of 40 mm in length from the circle of Willis to the mid-cervical point. All other MRI scans covered the full brain region. Single-PLD PCASL, multi-PLD PCASL (3 PLDs), and VSASL scans were performed at the same time as the PET scans (Guo et al., 2015). The ASL sequences were performed in random order to avoid the systematic bias due to the effect of ACZ on CBF over time. For all ASL scans, a proton density image (M_0)_ and a coil sensitivity map were acquired with a saturation recovery acquisition using TR = 2000 ms and other readout parameters identical to the ASL sequence. Acquisition parameters for all the ASL sequences are given in Table 1. For the multi-PLD PCASL sequence, crushing gradients (*V*_*ENC*_ = 4 *cm*/*s* as recommended by the ASL white paper (Alsop et al., 2015)) were included to exclude the signal in the arterial component before the 3D spiral readout. As shown in Fig. 1 A, PC MRI was performed before and 15 min after the administration of acetazolamide to measure the total flow volume and the velocity at the labeling plane of PCASL. The imaging slice of the PC MRI and the labeling plane of PCASL were planned perpendicular to the vessels found in the MRA scan and between C2 and C3 vertebrae. A T1-weighted structure image was acquired using a fast spoiled gradient echo T1-weighted scan (GE Brain Volume imaging, BRAVO) (Ellingson et al., 2015).

### 2.4. Flow volume and velocity quantification

PC MRI data were used to estimate the total flow volume and velocity before and after the administration of acetazolamide. For each PC MRI data, ten cardiac phases were acquired. The data were processed using Arterys software (Arterys Inc., San Francisco, CA, USA). The image of the last cardiac phase was used to segment the internal carotid and vertebral arteries. The flow volume and velocity of the arterial blood was averaged over all the cardiac phases for each inflow artery.

### 2.5. T1 relaxation of arterial blood and inversion efficiency quantification

The T1 relaxation of the arterial blood of each subject was quantified using the hemoglobin level, hematocrit, and blood oxygen saturation data (Hales et al., 2016). The detailed description of the quantification method can be found in Supplementary Materials. The labeling efficiency of the flow-driven inversion in PCASL was simulated in Matlab (The Mathworks, Inc., Natick, MA, USA, 2019) by solving the Bloch equations using matrix representation (Dai et al., 2008). “Unbalanced” PCASL settings were consistent with the labeling train parameters implemented in the in vivo studies given in Table 1. Homogenous field at the level of labeling plane was assumed. Simulations were performed for flow velocity values ranging from 3 to 100 cm/s with 1 cm/s step size assuming laminar flow (Maccotta et al., 1997). For each subject, the mean velocity of the left and right carotid arteries and left and right vertebral arteries was computed from the acquired PC MRI data. The effective labeling efficiency of each subject was computed by summing the labeling efficiency of each vessel and weighted by the fraction of blood volume contributed by each vessel out of the total volume (Vaclavu et al., 2018).

### 2.6. CBF quantification from ASL data

For the PCASL data, relative CBF was quantified using the model-fitting and spatially variational Bayesian inference techniques implemented in the FSL tool BASIL (Chappell et al., 2009; Groves et al., 2009). For the VSASL data, voxel-wise CBF values were estimated by fitting the ASL difference data to the model developed by Wong et al. (Wong et al., 2006). Absolute CBF of all ASL techniques was quantified using the proton density data (M_0)_ and the estimated relative CBF with coil sensitivity correction. Due to the short TR of the proton density data, the signal intensity was divided by $1-e^{-TR/T_{1,\;\text{Tissue}\;}}$, to estimate the equilibrium magnetization of the tissue, assuming *T*_1 *,Tissue*_ to be 1300 ms for the whole brain (Alsop et al., 2015). The corrected proton density data was then used to compute a calibration image, i.e. the equilibrium magnetization of the arterial blood, assuming a blood-brain partition coefficient (*λ*) of 0.9. The partial volume effects on the edge of the calibration image were corrected using the erosion and extrapolation (M.Y. Zhao et al., 2017). Finally, the CBF maps in absolute units (ml/100 g/min) were computed using the relative CBF and the calibration data.

### 2.7. CVR quantification

Two experiments were performed to compare the CVR estimated by PET and ASL to investigate the impacts of labeling efficiency and T1 relaxation of the arterial blood. In both experiments, the CVR of PET were used as the reference for comparison as shown in Fig. 1 B. The two experiments differed in whether the labeling efficiency and T1 of arterial blood were assumed using global values for the whole cohort or estimated for individual subjects as shown in Fig. 1 C and Fig. 1 D.

#### Experiment 1:

CVR and CBF of the ASL techniques were measured using the same labeling efficiency (85%) and T1 of arterial blood values (1.65 s) suggested in the ASL consensus paper for all subjects (Alsop et al., 2015).

#### Experiment 2:

CVR and CBF of the ASL techniques were measured using the estimated labeling efficiency and T1 of arterial blood values for each individual.

For both experiments, CBF in standard space was used to quantify voxel-wise CVR defined by the following equation:

$$CVR=\frac{CBF_{\text{post}-ACZ}-CBF_{pre-ACZ}}{CBF_{pre-ACZ}}\times 100\text{\%}$$

A robust range of CVR values was obtained by excluding the voxels considered outliers (outside 1st and 99th percentiles). The mean CVR of the whole brain and ROIs were computed.

### 2.8. Analysis masks and ROIs

A full brain mask for group analysis was created by extracting the brain from the T1-weighted template in the Montreal Neurological Institute (MNI)152–2 mm space using the FSL tool BET (Smith, 2002). A gray matter (GM) mask was created by (1) retaining the voxels with GM probability higher than 50% of the probabilistic atlas in MNI152–2 mm space (Mazziotta et al., 2001) and (2) intersecting with the full brain mask. The white matter (WM) mask was constructed using the same approach. Five other ROIs (frontal lobe, temporal lobe, occipital lobe, parietal lobe, and insula) were defined based on the Harvard-Oxford cortical and subcortical structural atlases (Desikan et al., 2006). All CBF and CVR images were transformed to the MNI-152 space by using rigid-body transformation of the ASL label and control difference or PET data to the T1-weighted structural image of each subject with FLIRT; followed by a second nonlinear transformation registering the T1-weighted image to the MNI152 2 mm standard brain using FNIRT (Woolrich et al., 2009).

### 2.9. Statistical analysis

For both experiments, the relationship between the individual CVR measured by PET and ASL was assessed by Pearson correlation coefficient analysis. Two-tailed paired t tests were performed to compare the CBF, ATT, and CVR in each ROI between different ASL techniques and PET. The normality of the data was checked using Kolmogorov-Smirnov tests before conducting the *t*-test (Smirnov, 1948). For the voxel-wise comparison, we performed permutation-based nonparametric tests with threshold-free cluster enhancement method that has shown better sensitivity over a wide range of data types and SNR values (Smith and Nichols, 2009). Before each statistical test, all images were smoothed using a Gaussian spatial filter of 3 mm FWHM. The statistical tests were conducted using the FSL tool RANDOMIZE with 100,000 permutations (Nichols and Holmes, 2002; Winkler et al., 2014) under the null hypothesis that the CBF or CVR values were the same. The family-wise error rate due to multiple comparisons was corrected using the method developed by Holmes et al. (Holmes et al., 1996), and the corrected p value was recorded and thresholded at 0.05 for significance. Voxel-wise CBF and CVR differences in standard space were computed for each test.

## 3. Results

### 3.1. Summary of subject information and experimental parameters

As shown in Table 2, there were 19 subjects (8 males, 37 ± 12 years) recruited in this study. The mean and standard deviation of the hemoglobin and hematocrit levels of the cohort were 14.7 ± 1.9 g/dL and 43 ± 5% respectively. Based on these values, the estimated T1 relaxation of the arterial blood of the group was 1.75 ± 0.07 s. During the PET/MRI experiment, 862 ± 123 MBq of ^15^O-water was injected, and 0.96 ± 0.09 g of ACZ was administered on average to each subject.

### 3.2. CBF and CVR results

**Fig. 2.**
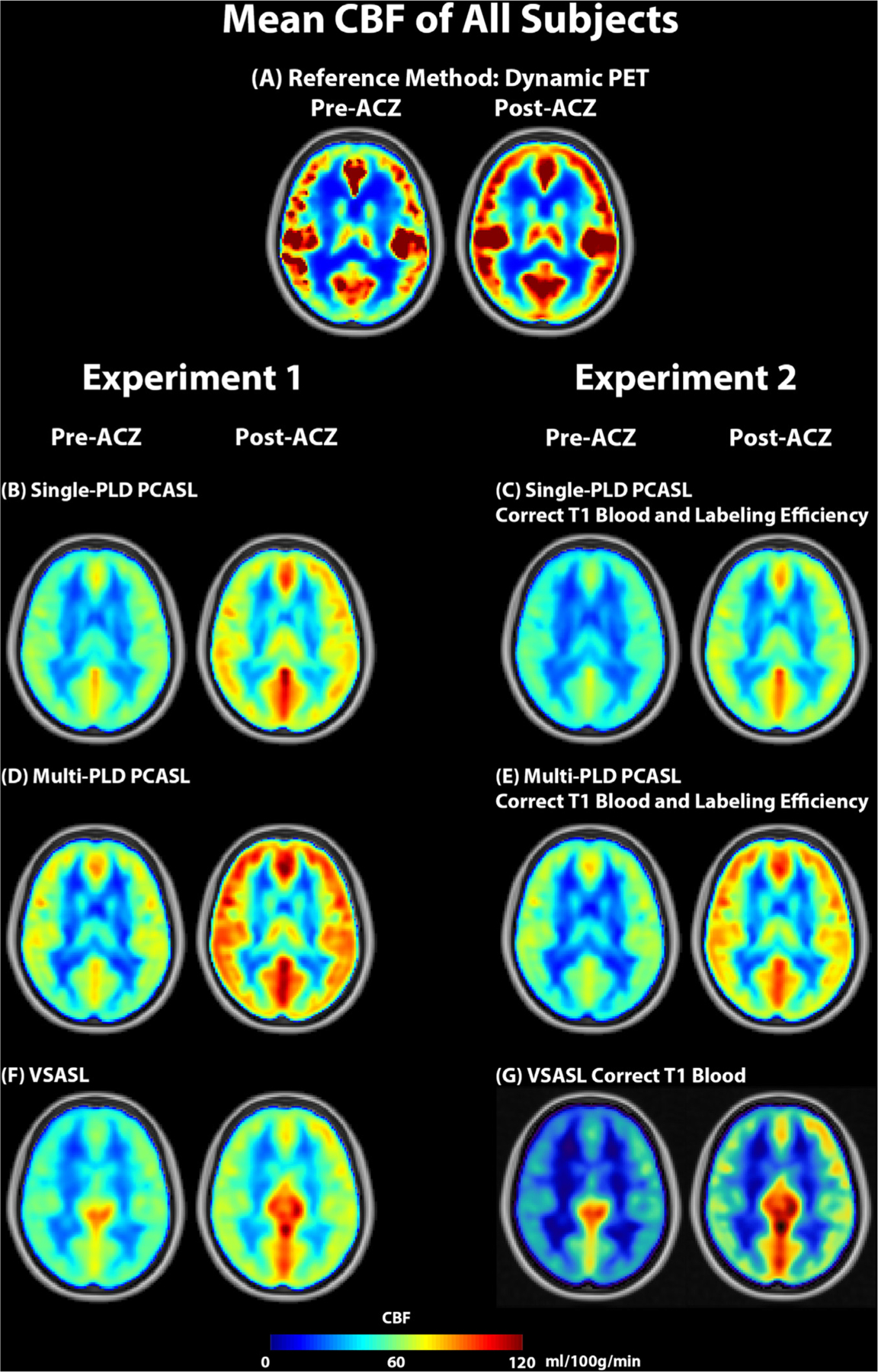
Mean CBF of all subjects pre and post-ACZ in both experiments. All imaging modalities captured significant CBF increase after the administration of ACZ.

**Fig. 3.**
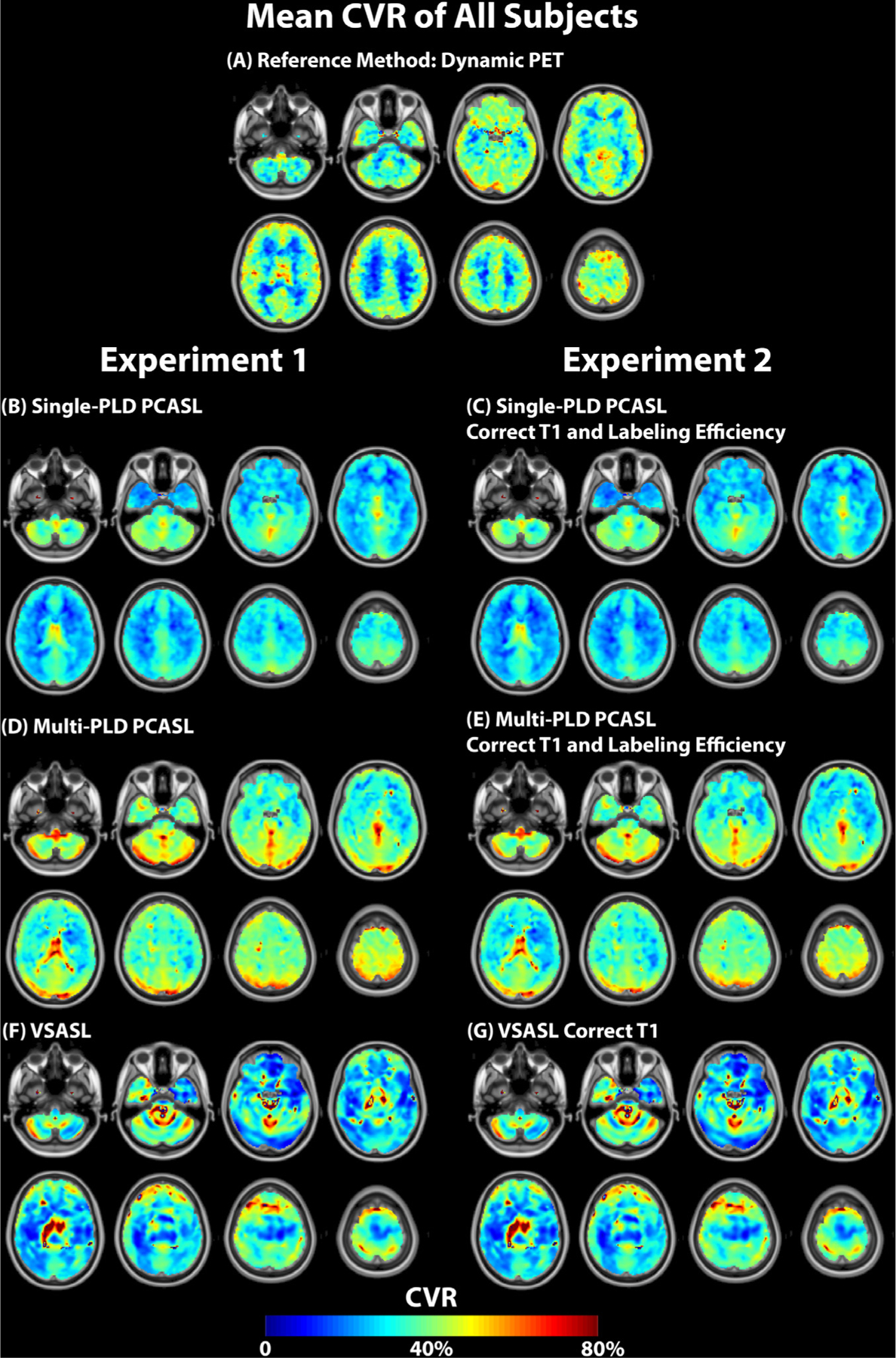
Mean CVR maps of all subjects using PET and MRI techniques in both experiments. For both single and multi-PLD PCASL methods (i.e., with and without individual corrections), the CVR of multi-PLD PCASL was higher than that of single-PLD PCASL. The effects of correcting T1 of blood and labeling efficiency on CVR were small. For VSASL, the mean CVR levels of the group were not significantly different before and after correcting for the individual’s arterial blood T1.

**Fig. 4.**
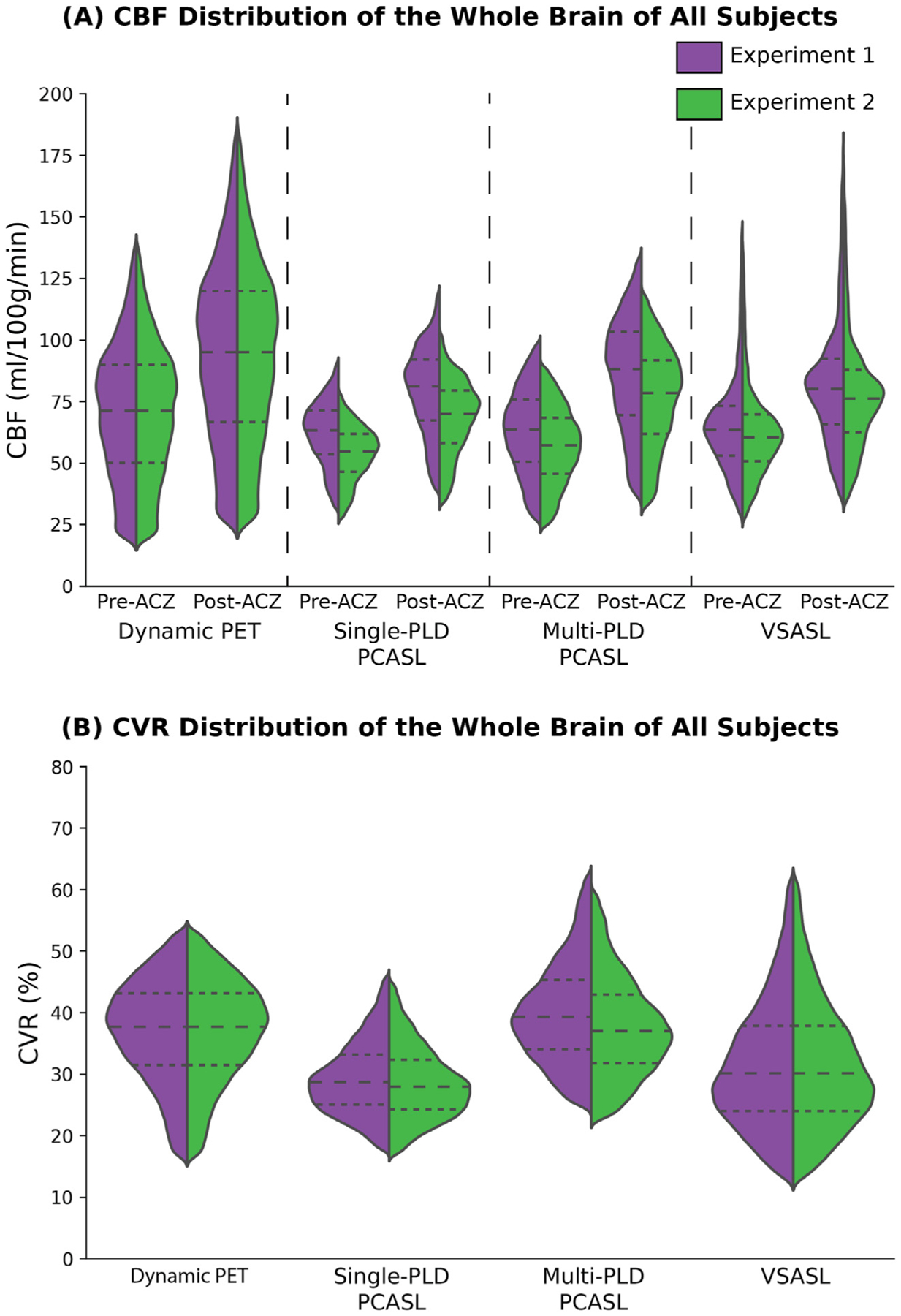
(A) CBF distribution of the whole brain of all subjects pre- and post-ACZ. CBF in experiment 2 were lower than the data in experiment 1 in all techniques. (B) CVR distribution of the whole brain of all subjects. Each dashed line of the violin plot indicates, from top to bottom, the 75th, 50th, and 25th percentile.

Fig. 2 shows the mean CBF maps of all subjects pre and post-ACZ using PET and ASL techniques in both experiments. Fig. 3 shows the mean CVR maps of all subjects using PET and ASL techniques in both experiments. Figure S1 and Figure S2 show the CBF and CVR maps of an example subject (male, 30 years). Fig. 4 shows the CBF and CVR distribution of the whole brain of all subjects. Overall, all imaging techniques captured the significant CBF increase induced by ACZ.

### 3.3. Global and regional CVR differences between PET and ASL

**Fig. 5.**
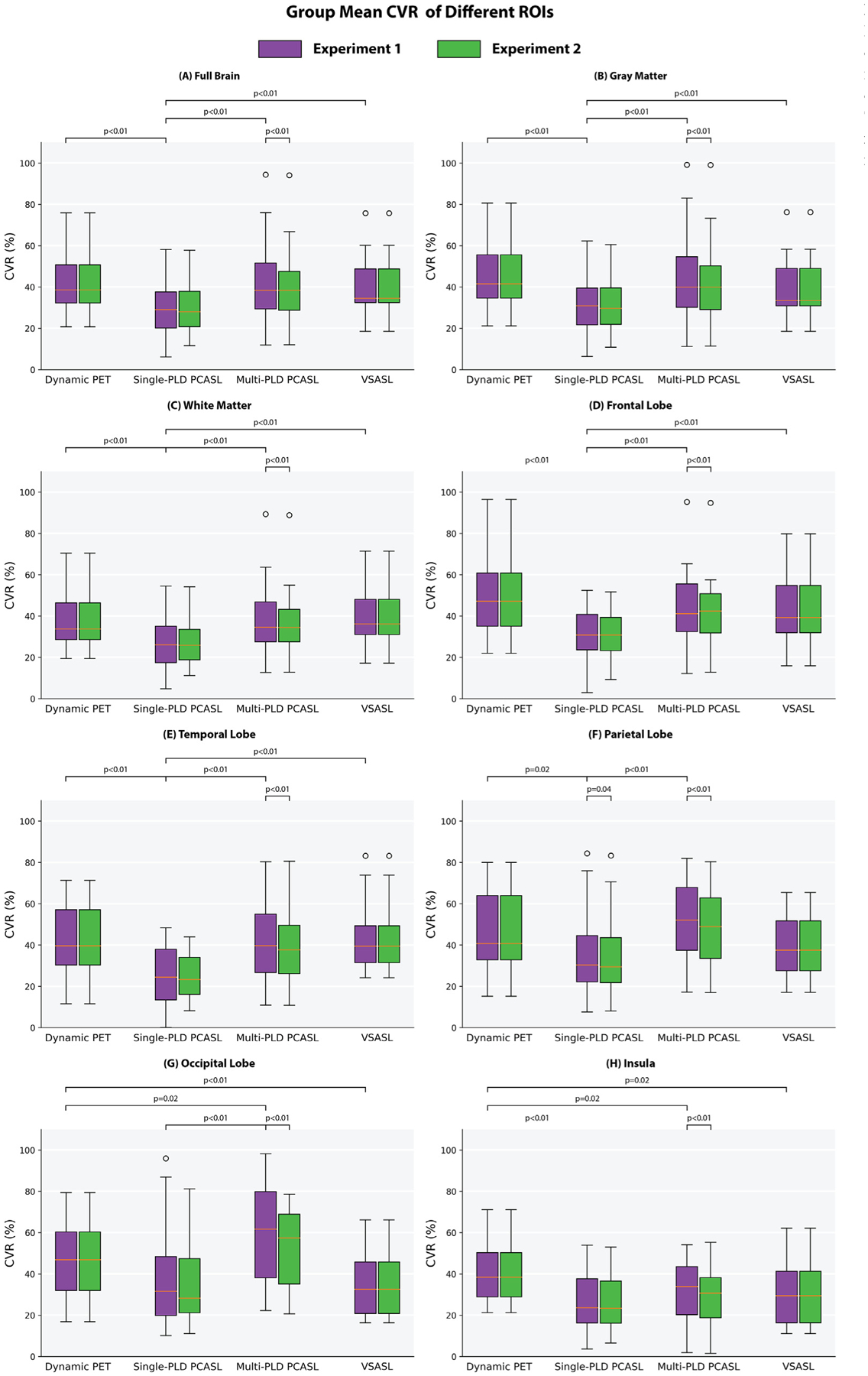
Mean CVR of all subjects in each ROI and the statistical test results. The CVR of Dynamic PET and VSASL were identical in both experiments. Each box plot indicates, from top to bottom, the maximum, 75th, 50th, 25th percentiles, and minimum not considering outliers, and the outliers represented by open circles.

The p-values of Kolmogorov-Smirnov tests were all greater than 0.05, indicating that our data did not differ significantly from the one normally distributed. Fig. 5 shows the group mean CVR in different ROIs in the two experiments. The CVR of single-PLD PCASL was significantly lower than the CVR of PET in all ROIs except the occipital lobe, indicating that there was a systematic bias for single-PLD PCASL. In terms of the effects of correcting for T1 of blood and labeling efficiency for ASL data, although the CVR levels estimated by multi-PLD PCASL was significantly lower in Experiment 2, the effect size was less than 5%. This implied that despite the T1 of the arterial blood and labeling efficiency being statistically significant factors, they induced subtle effects on CVR quantification using multi-PLD PCASL. For the single-PLD PCASL technique, correcting for the T1 of arterial blood and labeling efficiency in Experiment 2 only changed CVR significantly in the parietal lobe. The CVR values of VSASL were similar in all ROIs and significantly different from the values of single-PLD PCASL.

**Fig. 6.**
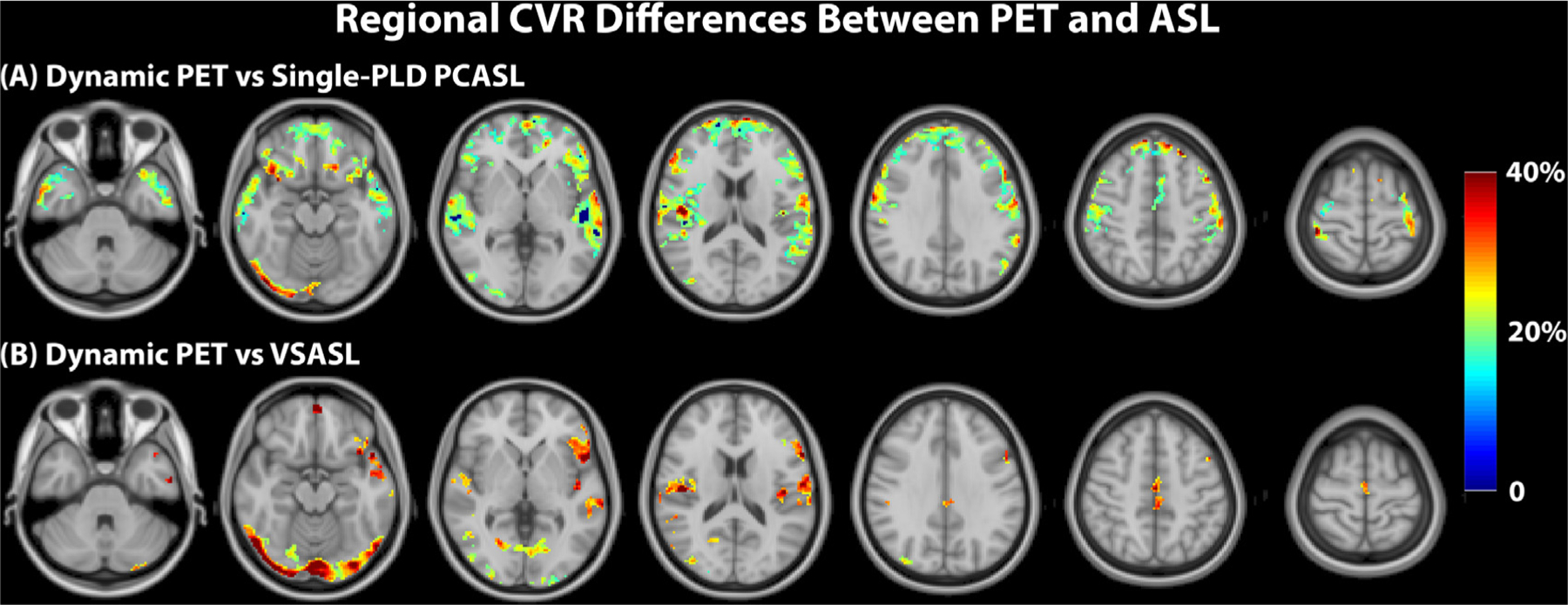
Regions of significant CVR differences (t-test; corrected p-value *<* 0.05) between PET and ASL techniques and effect size. No significant CVR differences were found between PET and multi-PLD PCASL (not shown).

**Fig. 7.**
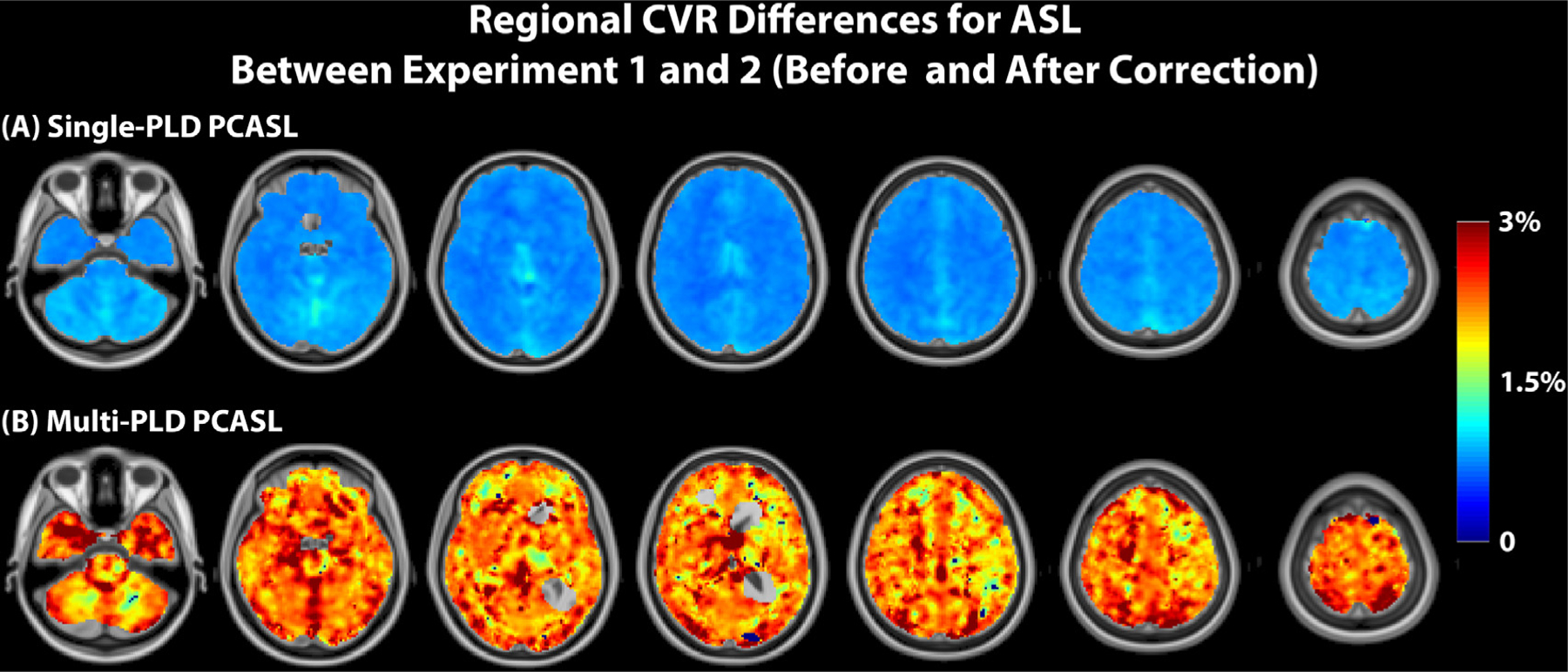
Regions of significant CVR differences (t-test; corrected p-value *<* 0.05) for PCASL before and after correcting for T1 of arterial blood and labeling efficiency and effect size.

Fig. 6 and Fig. 7 shows the results of the paired t tests and the regional CVR differences (with corrected p value *<* 0.05). For the comparison between PET and ASL, using single-PLD PCASL or VSASL for CVR quantification, the results were significantly lower than with PET by 30% in certain cortical regions. No regional differences were found between the CVR values of PET and multi-PLD PCASL and between Single-PLD PCASL and VSASL (not shown). For both single and multi-PLD PCASL techniques, correcting for the subject T1 of arterial blood and labeling efficiency only caused very a small CVR decrease of less than 5%. For the two PCASL techniques, the primary difference of CVR quantification clustered near the occipital lobe. The regional CVR differences between multiple and single PLD PCASL techniques can be found in Figure S3 of Supplementary Materials, and the regional CVR difference between PET and ASL after correcting for T1 blood and labeling efficiency can be found in Figure S4 of Supplementary Materials.

### 3.4. Correlation of CVR between PET and ASL

**Fig. 8.**
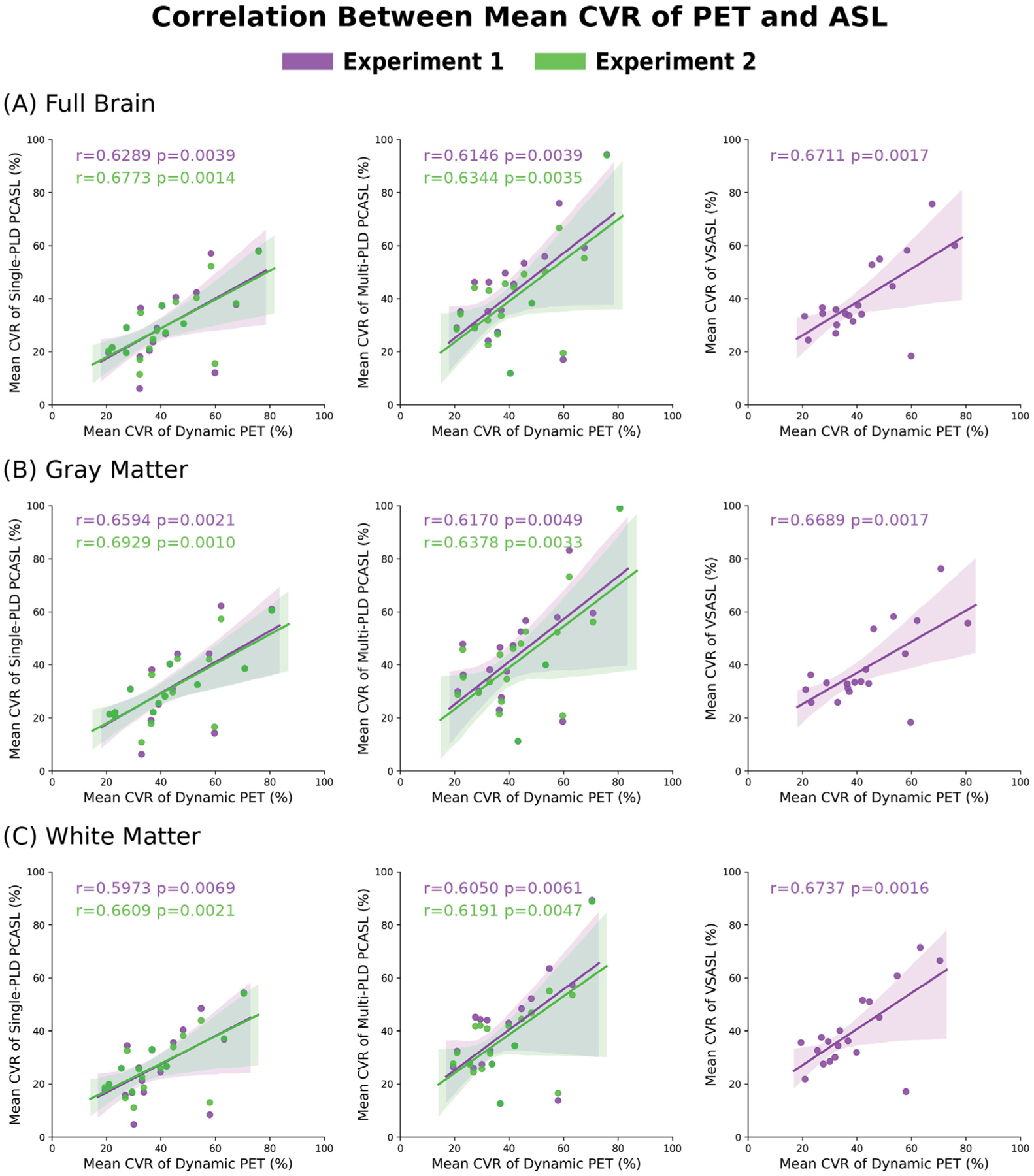
Correlation between the mean CVR measured by PET and ASL methods for the full brain, GM, and WM in both experiments. Overall, correcting for T1 blood and labeling efficiency slightly improved the correlation with PET CVR for both PCASL techniques. The correlation between PET and VSASL were identical in both experiments, hence only the results of Experiment 1 are displayed. The shaded area represents the 95% confidence interval.

Fig. 8 shows the correlation between the mean CVR of PET and ASL methods in the full brain, GM, and WM for both experiments. Table S1 and Table S2 of Supplementary Materials show the slopes of the fitted regression lines. In general, correcting for the T1 blood and labeling efficiency improved the correlation between the mean CVR of PET and PCASL by 8% for single-PLD PCASL and 3% for multi-PLD PCASL for the full brain. Figure S5 of Supplementary Materials shows the correlation plots for other ROIs. For single-PLD PCASL, the region with the highest CVR correlation with PET was in the temporal lobe; for multi-PLD PCASL the mean CVR in the frontal lobe demonstrated the highest correlation with PET.

### 3.5. Changes following vasodilation

**Fig. 9.**
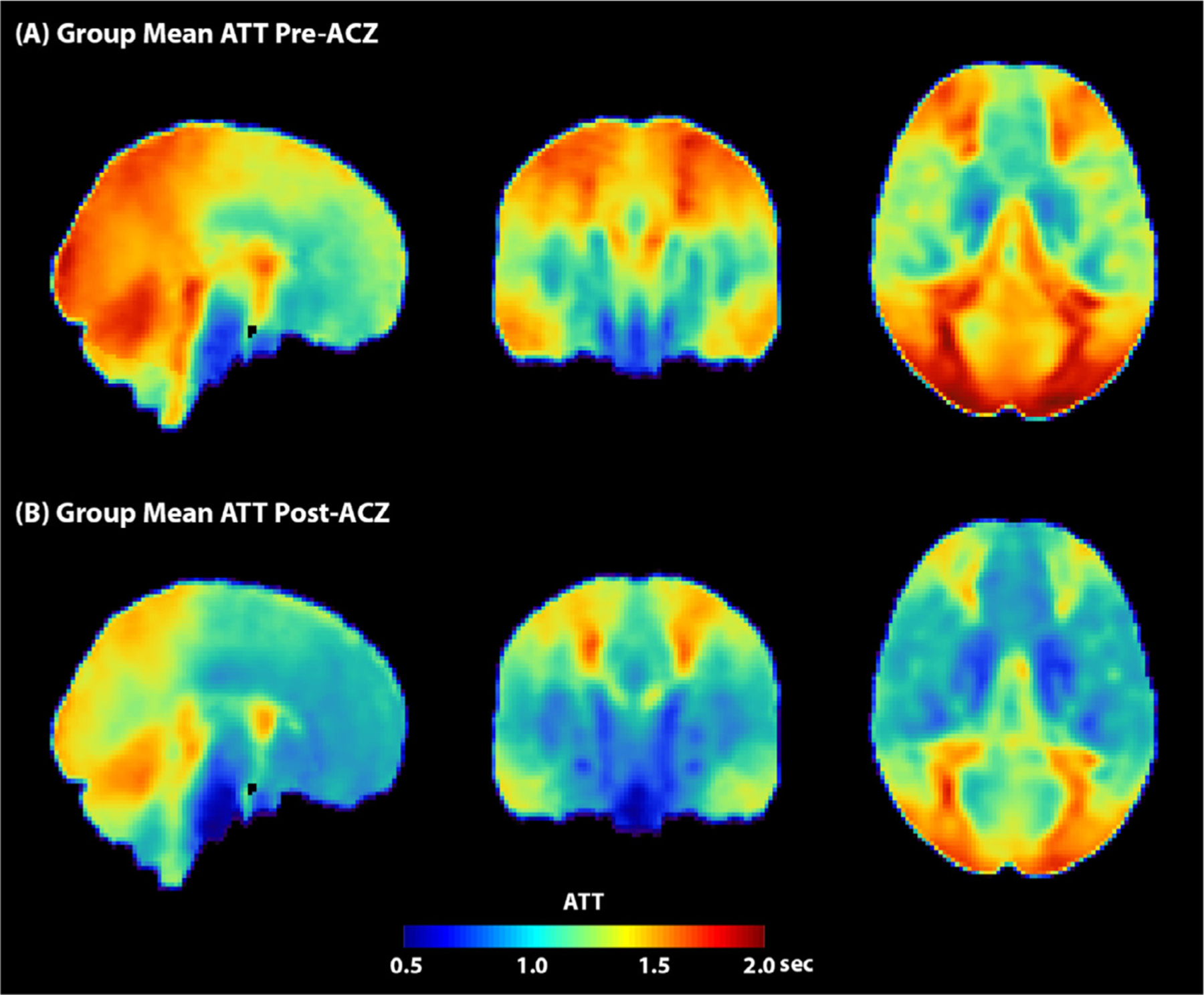
Group mean ATT maps of multi-PLD PCASL data. Overall, the ATT decreased in all regions after the administration of ACZ. For both pre- and post-ACZ conditions, the ATT of the posterior region was higher than other regions of the brain.

**Fig. 10.**
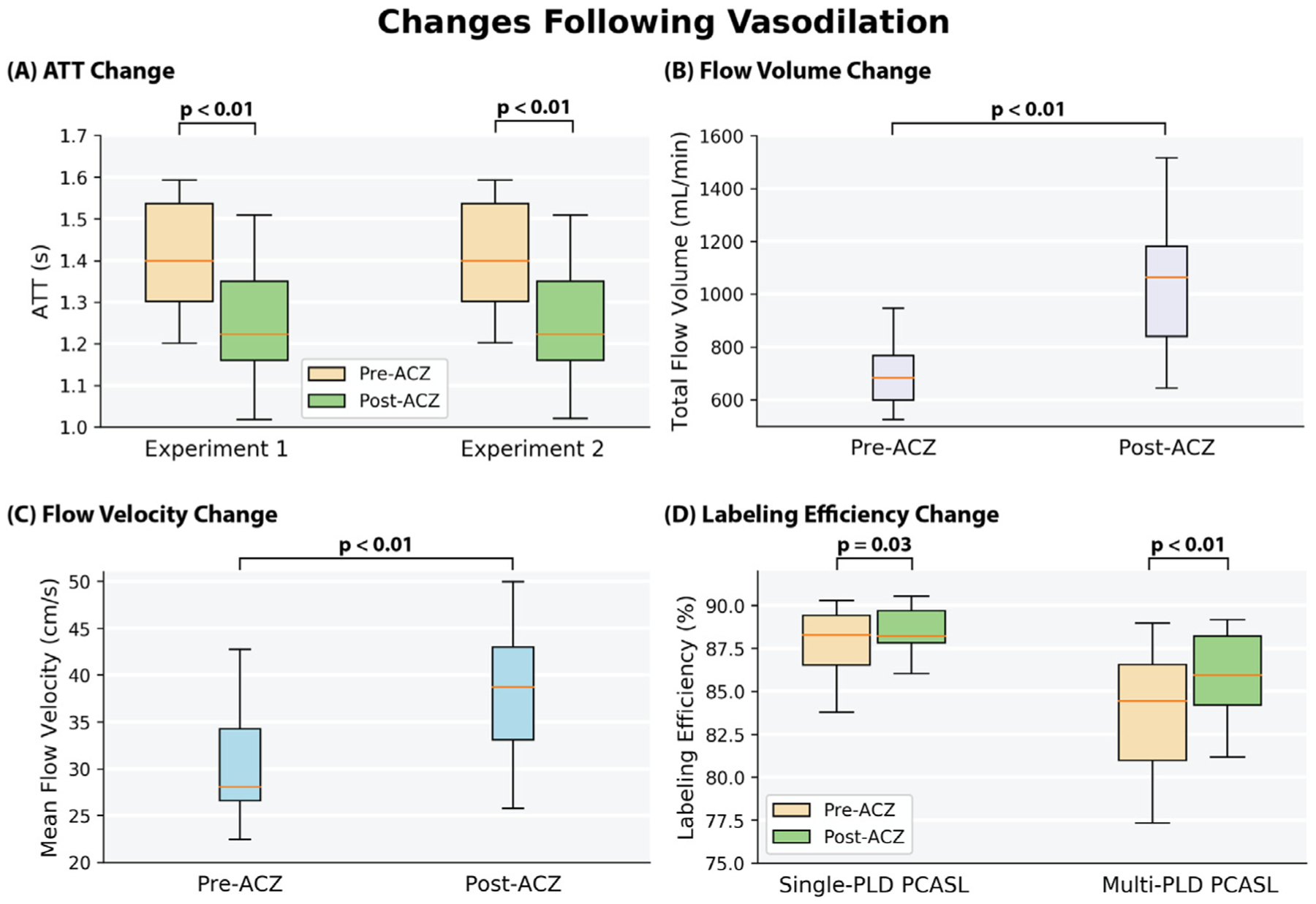
(A) The mean ATT of the whole brain measured by multi-PLD PCASL was reduced significantly after the administration of ACZ in both experiments by about 200 ms. (B) The total flow volume of the carotid and vertebral arteries and (C) their mean velocity measured by PC MRI increased significantly after the administration of ACZ. (D) The labeling efficiency of the group increased significantly for both single and multi-PLD PCASL after the administration of ACZ. Each box plot indicates, from top to bottom, the maximum, 75th, 50th, 25th percentiles, and minimum.

Fig. 9 shows the group mean ATT maps before and after the administration of the vasodilator ACZ. Figure S6 shows the ATT map of an example subject (male, 30 years). Fig. 10 shows the mean ATT of the whole brain estimated by multi-PLD PCASL, changes in total flow volume and flow velocity in the carotid and vertebral arteries measured by PC MRI, and the labeling efficiency change for single and multi-PLD PCASL in the pre- and post-ACZ conditions. The flow velocity and labeling efficiency of different arteries can be found in Figure S7, Figure S8, and Figure S9 of Supplementary Materials. Overall, the administration of ACZ caused significant variations in all of these parameters. Specifically, the mean ATT of the whole brain of the group decreased significantly by 10% post-ACZ in both experiments. The increase caused by ACZ administration was 51 ± 23% for the total flow volume and 25 ± 19% for the mean flow velocity measured by PC MRI. From the Bloch equation simulations, this translated into small but significant increases in labeling efficiency post-ACZ (0.7 ± 1.2% for single-PLD PCASL and 2.4 ± 2.0% for multi-PLD PCASL).

## 4. Discussion

In this work, we investigated CVR measurements induced by ACZ using simultaneous ^15^O-water PET and ASL MRI on a healthy cohort. We applied various quantification techniques to measure and compare CBF and CVR based on global and regional differences, including dynamic PET, single-PLD PCASL, multi-PLD PCASL, and VSASL. Two experiments were conducted to assess the potential confounding factors affecting the ASL CVR measurements, including changes in ATT, the T1 relaxation of arterial blood, and the labeling efficiency of PCASL. Using the dynamic PET method as a reference, the primary findings of this study are (1) that the multi-PLD approach is the preferred PCASL technique for CVR quantification and the impacts of individual arterial blood T1 and labeling efficiency were small; (2) the single-PLD PCASL method underestimated CVR and post-ACZ CBF due to the significant reduction in ATT; and (3) VSASL is a feasible technique in measuring CVR of GM and insensitive to variations in the arterial blood T1 relaxation but should be used with care due to the potential artifact near the ventricles.

### 4.1. CVR quantification using dynamic PET

Pharmacokinetic modeling of dynamic ^15^O-water PET data has been regarded as the reference standard for CBF quantification (Fan et al., 2016). Although this technique allows absolute CBF measurement with high accuracy and reproducibility, obtaining an accurate AIF using arterial blood sampling is challenging and has prevented its application on CVR studies. Here, the issue was partially resolved using an image derived AIF estimated from the MRA data and the arterial phase of the dynamic PET data, which enabled the quantification of the arterial blood volume and minimized the spill-in effect with an optimal dose of ^15^O-water (Khalighi et al., 2018). Using this approach, the resulting CBF and CVR were within the range of the values reported in previous studies that used the arterial blood sampling technique (Heijtel et al., 2014; Puig et al., 2019), implying that our image derived AIF method was suitable for CBF and CVR quantification using ^15^O-water PET. Although the CBF measured by the dynamic PET method demonstrated high between-subject variations for both pre- and post-ACZ conditions, the between-subject variation for CVR was substantially lower (Fig. 4). A possible explanation is that the capacity for CBF to reach to its maximum (i.e. CVR) of the entire cohort was considerably lower than the range of the CBF of all subjects. The ASL techniques also yielded a similar CVR pattern in which the between-subject variation of CVR was lower than that of CBF. Thus, these data indicated that the variability of CVR for healthy individuals is smaller than that of CBF.

### 4.2. Comparison between PET and ASL techniques

Using the CVR quantified by dynamic PET as a reference, we compared the CVR of PCASL and VSASL and the impact of correcting T1 of the arterial blood and labeling efficiency for each subject. Based on the paired t-test results, the multi-PLD PCASL technique was the most accurate ASL method both before and after incorporating the individual T1 of the arterial blood and labeling efficiency into the analysis. The group mean CVR of the multi-PLD PCASL technique was 40%, similar to the values quantified by PET (39%, Fig. 4 B). Similar results were found in a previous study by Puig et al. where CVR was also quantified using the ^15^O-water PET and multi-PLD PCASL techniques, despite the use of arterial blood sampling for PET and the multi-PLD PCASL method incorporating a larger number of individual PLDs than our implementation (Puig et al., 2019). The differences between the present study and the previous work included: (1) we applied a relatively shorter bolus duration in the multi-PLD PCASL sequence (1700 vs 1800 ms), resulting in a shorter acquisition time (4:47 vs 7 min); (2) our sequence included crushing gradients to remove the signal contamination in the arterial blood while the previous study did not; (3) we use a slightly higher temporal resolution in the dynamic PET reconstruction (30 × 1, 10 × 3, 12 × 5, 12 × 10, 12 × 30 vs 18 × 5 s, 9 × 10 s, 4 × 15 s); (4) the attenuation correction of the present study was achieved using a two-point Dixon 152 MRI acquisition and an atlas based algorithm while the study by Puig et al. used μ-maps generated with a separately acquired low-dose CT scan. In our data, no significant regional differences were found between the CVR of dynamic PET and multi-PLD PCASL, making multi-PLD PCASL a preferred candidate to replace dynamic PET for the CVR measurements of healthy subjects. By contrast, the CVR measured by single-PLD PCASL was significantly lower than PET (by 3 – 12% in different ROIs, Fig. 5). Correcting for the T1 of blood and labeling efficiency variations caused little change to the overall results. Our comparison of single and multi-PLD PCASL vs dynamic PET indicated that the variation in ATT was the principal factor for the more accurate CVR measurement using PCASL techniques. The CVR differences between PET and Single-PLD PCASL or VSASL, seen in Fig. 6, appeared to cluster at the vasculature. The arterial suppression techniques were not used for the single-PLD PCASL sequence because the implementation aimed to comply with the recommendations of the ASL white paper and to allow all 3 ASL sequences to be acquired simultaneously with the PET data within 15 min. For VSASL, the velocity fluctuation and/or the in-plane flow possibly reduced the effectiveness of the vascular crushing in these regions with big vessels. Consequently, there might be labeled blood water in the vasculature that affected the precise quantification of CBF and CVR, leading to the regional differences seen in Fig. 6. When comparing the individual CVR levels measured by PET and ASL, the correlation of Experiment 2 was greater than Experiment 1 in all ROIs. This implied that making individual corrections for hematocrit and blood velocity enhanced the correlation for all regions of the brain (Fig. 8 and Supplementary Materials Figure S5). Comparing between different ASL techniques in each experiment, however, we obtained mixed results for the ASL technique with the highest correlation with PET in each ROI. For example, CVR of multi-PLD PCASL showed the highest correlation for the frontal lobe (*r* = 0.64) while single-PLD PCASL was the highest for the parietal lobe (*r* = 0.54) in Experiment 1.

The PET and ASL acquisition techniques used in this work were selected based on (1) they are widely available in most GE PET and MRI scanners and (2) their acquisition time (about 5 min) matches the ones used for clinical applications. This ensures the generalization of the data performed on other scanners by the same vendor. The different resolutions between the PET and ASL data may cause partial volume effects, which only influence tissue specific CBF/CVR measurements. The different label/control pairs in ASL acquisition may affect the SNR of the ASL data. However, their acquisition time was similar and we applied an analysis method based on Bayesian inference that has shown high robustness to different SNRs as well as data collected by different ASL techniques, allowing a high accuracy in the CBF and CVR quantification for this cohort (Chappell et al., 2009), (Zhao et al., 2020; Chappell et al., 2013; Pinto et al., 2020). Therefore, the different acquisition techniques might alter some regional CVR values, but not the conclusions related to the comparison between ASL and PET.

Although the mean CVR maps showed difference across acquisition strategies, little or no regional differences were observed after performing the voxel-wise statistical test. Specifically, the standard deviation of the full brain CVR for each imaging modality were 19% (PET), 12% (Single-PLD PCASL), 17% (Multi-PLD PCASL), and 16% (VSASL) for this cohort. Previous studies reported a broad range of CVR in normal subjects (Zhao et al., 2020), (Leung et al., 2016; Boles Ponto et al., 2004; Lipp et al., 2015), which indicated that either the reproducibility of the measuring technique was poor or there is a genuinely large variability among individuals. This open question may be addressed by extending the current work to investigate the reproducibility of different CVR techniques in a test-retest study.

Based on the inter-modality CVR comparison between PET and ASL MRI, we can conclude that, using PET as a reference, the multi-PLD PCASL should be the favorable technique due to its high accuracy in mean CVR quantification while the single-PLD PCASL and VSASL underestimate CVR in certain regions. Care should be taken when using VSASL in CVR quantification due to the potential artifact near the ventricles. Applying individual corrections for hematocrit and flow velocity should be considered due to its positive influence on the correlation of ASL-based CVR measurements with PET, though the absolute magnitude of these changes are small.

### 4.3. Comparison between ASL techniques

In measuring the CVR levels using PCASL, we applied two techniques: the single-PLD PCASL sequence (available in all GE scanners) and a multi-PLD PCASL sequence with 3 PLDs. The single-PLD PCASL technique has been widely applied in several studies and demonstrated high robustness in CBF quantification at a resting condition (Mutsaerts et al., 2020; R.M.E. Steketee et al., 2015; R.M.E. Steketee et al., 2015). The multi-PLD PCASL technique used Hadamard encoding technique in the labeling module and achieved a high reproducibility in CBF and ATT measurement for healthy subjects in our previous work (Guo et al., 2018). Our previous work showed that the 3-PLD PCASL technique showed that the 3-PLD acquisition was more accurate in estimating ATT in simulation mainly due to improved SNR in ASL signals at individual PLDs and there was no significant CBF differences measured in vivo (Guo et al., 2017). Although the GM ATT estimated using 3 and 7 PLDs had significant differences after Bonferroni correction, the effect size was less than 2%, which was substantially lower than the ATT increase (10%) induced by the vasodilator in this study. To improve the accuracy of model-fitting, we also incorporated a spatially regularized Bayesian framework that attempts to estimate both CBF and ATT in every voxel, with each being subject to a prior distribution on the value derived from the estimated values in the neighboring voxels (Groves et al., 2009). In addition, the acquisition time of the 3-PLD PCASL was slightly shorter than the 7-PLD PCASL to allow 3 ASL sequences (single-PLD PCASL, multi-PLD PCASL, and VSASL) to be acquired with the PET scan within 15 min. No significant difference in root-mean-square error (RMSE) was found in the analysis that compared the accuracy of simulated 3-PLD and 7-PLD PCASL data, as shown in Table S3 and Figure S10 in Supplementary Materials, making the 3-PLD acquisition a suitable method for CBF and CVR quantification. Nevertheless, a systematic comparison, similar to the study by Woods et al. (Woods et al., 2020), may be desired to determine the optimal number of PLDs for CVR quantification using multi-PLD PCASL. Although the two PCASL techniques produced similar CBF levels at baseline (pre-ACZ), the CBF estimated by the multi-PLD PCASL technique was significantly higher post-ACZ, which is shown qualitatively in Fig. 2 and quantitatively in Fig. 4. The reason was that the single-PLD technique was unable to compensate for the ATT variations induced by the vasodilator while the multi-PLD technique revealed a significant drop in ATT by 10% post-ACZ (Fig. 10 A), consistent with previous studies (Zhao et al., 2020), (Federau et al., 2017). These data validated the assumption that the single-PLD PCASL approach, despite being accurate for CBF measurements at baseline conditions using a long PLD, have limitations for CVR quantification, leading to an underestimation between 14% and 30% in different ROIs of this cohort (Fig. 5). The ATT maps revealed a well-defined spatial variation that the occipital lobe had longer ATT than other regions for both before and after the administration of acetazolamide conditions (Fig. 9). Assuming a mean ATT of 1.5 s in the occipital lobe as shown in Fig. 9 B, the single-PLD PCASL underestimated CBF by 17% for this region after the administration of acetazolamide, making the single-PLD a less accurate technique for CVR measurements. In a recent study on the optimal design of ASL sequences, the multi-PLD PCASL technique was recommended as the most accurate method for both CBF and ATT quantification (Woods et al., 2020). Our data not only supported this argument with in vivo data but also demonstrated the effect size of CBF and ATT changes when the physiology of the subject was altered by a vasodilator.

Comparing the post-ACZ CBF between the single and multi-PLD PCASL methods, a discrepancy of 12% was found for the whole brain (Fig. 4 A). Specifically, the CVR levels were significantly higher in the posterior regions of the brain, where the perfusion was supplied by the vertebral arteries and the ATT exhibits the largest variations. These findings contrast with observations in another study that investigated the impact of ATT on CVR by comparing the single-PLD PCASL and multi-TI pulsed ASL approaches (Zhao et al., 2020). A possible explanation is that the current work applied different labeling and imaging modules from the previous study for the single-PLD PCASL sequence. For example, the labeling duration implemented here was 1450 ms in comparison with 1800 ms; the PLD used here was 2025 ms in contrast with 1800 ms. Such variations may affect the CBF quantification post-ACZ due to the significant increase in flow velocity induced by ACZ, as shown in Fig. 10 C. Therefore, it is important to control for ATT to obtain accurate CVR quantification using PCASL.

VSASL was developed to eliminate the impacts of ATT and labeling efficiency variations on CBF quantification by using robust non-spatially selective pulses to label the arterial blood and choosing an optimal cutoff velocity (Vc) in both labeling and imaging modules (Wong et al., 2006; Guo et al., 2015). The current work is the first application of VSASL on CVR quantification for a healthy cohort. The CVR values estimated by VSASL were within the range of both PET and PCASL (between 20% and 60%, Fig. 5), making the VSASL a potentially viable technique for examining the whole brain CVR of healthy subjects. The distribution of the voxel-wise CVR and CBF of VSASL (Fig. 4), however, demonstrated the largest range among all the PET and ASL techniques. This was chiefly caused by the signal contamination near the ventricles that resulted in an overestimation in CBF as shown in Fig. 2. This artifact is typically attributed to the diffusion sensitivity of the labeling pulse, resulting in errors in the CSF region after subtracting the label and control images (Wong et al., 2006). A detailed description of the velocity profiles and diffusion attenuation effects can be found in Supplementary Materials. Although the problem may be mitigated by increasing the *Vc* value or applying a T2-FLAIR module before imaging (Qin and van Zijl, 2016), the CBF would be more vascular-weighted with lower SNR that requires a longer acquisition time. Other sources of error may include the influence of cardiac pulsatility and the blurring effects of the 3D spiral readout, which would potentially manifest in different ways for VSASL.

Due to the low resolution, ASL is limited by partial volume effects that affect the precise quantification of CBF (Asllani et al., 2008; Chappell et al., 2011; Donahue et al., 2006. Although several correction techniques have been developed, their impacts were primarily on the measurement of tissue specific CBF (such as gray matter CBF), according to a systematic study on the various partial volume correction techniques (M.Y. Zhao et al., 2017). Although performing the correction is possible using partial volume estimates derived from the T1-weighted structural image, this might only alter some of the CBF values noted in this study, but not the conclusions related to comparisons between different ASL techniques and PET. Although performing partial volume correction affects the CVR of specific tissues, the actual impact on the reginal CVR differences between ASL and PET remains an open question, which will potentially manifest in different ways in the quantification of CVR using different ASL techniques. In this work, we focused on comparing the CVR quantification using different ASL techniques and impacts of transit time and blood characteristics. However, we did not seek to include gray matter or white matter CVR differences, which might be an interesting direction to investigate in future work.

From the CVR results estimated by the ASL data, we can conclude that controlling the for variations in ATT is significant in PCASL-based CVR quantification and that VSASL can be applied to measure CVR levels of GM regions accurately.

### 4.4. Impacts of blood T1 and labeling efficiency

The data in Experiment 2 supported our hypothesis that the variations in the T1 relaxation time of arterial blood and labeling efficiency would significantly affect the CVR measurement using PCASL. Since the T1 of blood of each subject was computed using the hematocrit value as shown in the equation in Supplementary Materials, these two parameters were considered together in Experiment 2. According to the general kinetic model, the labeling efficiency and T1 relaxation time of arterial blood affect the CBF and CVR quantification of the whole brain globally (Buxton et al., 1998). The T1 of the arterial blood of the group (1.75 s), measured using the blood test results, was significantly higher than the default value (1.65 s) suggested by the ASL consensus paper (Alsop et al., 2015). According to the general kinetic model, such a difference would underestimate the CBF of PCASL by 4%, assuming a constant tissue T1 and ATT for the whole brain (Buxton et al., 1998). In terms of the labeling efficiency variations, the mean velocity in carotid and vertebral arteries changed from 27 to 38 cm/s, a significant increase of 41% (Fig. 10 C). From the Bloch equation simulations, such variations in flow velocity resulted in slightly increased labeling efficiency for both the single and multi-PLD PCASL techniques (4% and 5% respectively) (Fig. 10 D). Correcting for variations in blood T1 and labeling efficiency caused the CVR of the cortical regions to decrease by less than 1% for single-PLD PCASL (Fig. 7 A) and by 2% for multi-PLD PCASL (Fig. 7 B). In a systematic study that examined the impact of T1 blood and labeling efficiency on PCASL, the authors demonstrated that including the indirect measurements of these two parameters would reduce the errors of CBF quantification using simulated data (Bladt et al., 2020). Here, our ASL CBF results implied that correcting for blood T1 and labeling efficiency for individual subjects improved the correlation with the reference PET CBF (Fig. 8), which supported previous findings that controlling these two factors could benefit CBF and CVR measurements.

**Fig. 11.**
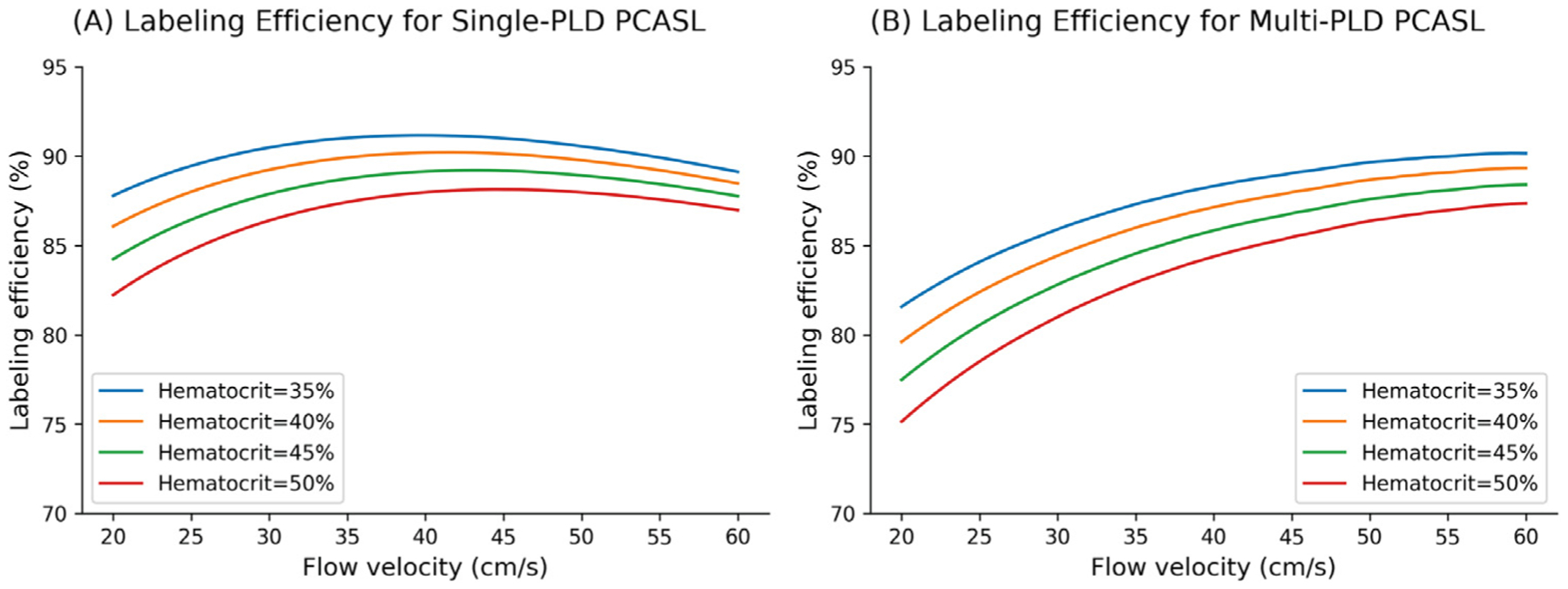
Changes in labeling efficiency for single and multi-PLD PCASL at different hematocrit and flow velocity levels. For both labeling techniques, the labeling efficiency increased slightly in the expected range of the flow velocity, though the magnitude of increase is higher for the multi-PLD implementation. Higher hematocrit resulted in a lower labeling efficiency at the same flow velocity.

The labeling efficiency of PCASL depends on factors associated with (1) the blood characteristics of the subject, such as hematocrit and flow velocity, and (2) the implementation of the labeling technique, such as the pulse duration, strength, and spacing (Dai et al., 2008). A common belief is that the labeling efficiency correlates negatively with the flow velocity, resulting in the labeling efficiency to decrease post-ACZ (Liu et al., 2018). In our implementation, however, the labeling pulses of both PCASL sequences were designed such that the resulting labeling efficiency was relatively insensitive to the expected range of the flow velocity and hematocrit of our cohort, as shown in Fig. 11. For example, the original PCASL labeling train used a mean and maximum gradient strength at 1 mT/m and 9 mT/m respectively (Dai et al., 2008). In our study, we reduced the values of these parameters to 0.7 mT/m and 7 mT/m respectively, allowing the labeling efficiency to remain consistent for a wider range of flow velocity levels. Consequently, the labeling efficiency only increased marginally by 3% post-ACZ and the resulting CVR decreased slightly by less than 1%. These data implied that, by carefully selecting the labeling parameters to accommodate the expected flow variations, CVR can be measured using PCASL without the impact of labeling efficiency changes.

In this work, simulations of labeling efficiency assumed a perfect homogenous field at the level of labeling. Although off-resonance effects decrease labeling efficiency (Shin et al., 2012), (Jahanian et al., 2011), these off-resonance effects were identical for both scans and therefore are expected to have similar effects on labeling efficiency before and after vasodilation. We assumed laminar flow in all internal carotid and vertebral arteries. An alternative approach is assuming pulsatile flow in the labeling efficiency estimation, as demonstrated in a study that used RR cycle interval velocity profile and calculated contributions of different velocities as the flow-weighted average across the velocity distribution function (L. Zhao et al., 2017). The most influential factors on PCASL labeling efficiency were selecting an unbalanced scheme and placing the labeling plane between C2 and C3 (Jahanian et al., 2011), (L. Zhao et al., 2017), both of which were adopted in our study. In improving the accuracy of labeling efficiency quantification, we also used a mean velocity of the 10 cardiac phases in the PC MRI data, which improved the overall SNR and reduced potential errors due to the manual segmentation of the vessels. The labeling techniques used in this study have been implemented in the product ASL (single-PLD PCASL) and eASL (multi-PLD PCASL) sequences of GE systems. Thus, the conclusion holds for all the PCASL data collected using GE’s 3T MRI systems.

For VSASL, the variation in T1 of arterial blood was the primary factor affecting CBF quantification because VSASL applied a pulsed labeling technique, which is insensitive to the variations in flow velocity pre and post-ACZ (Guo et al., 2015). According to the CBF quantification model for VSASL described in Supplementary Materials, the variation in T1 of arterial blood should have the same impact for both pre- and post-ACZ CBF measurements (Wong et al., 2006). Since the CVR was computed using the ratio between the CBF changes and baseline CBF, however, changes in T1 of arterial blood has no impact on the CVR quantification, as shown in Fig. 2 and Fig. 3. Thus, CVR measured by VSASL is insensitive to either blood T1 or flow velocity variations.

Based on presented results, we can conclude that incorporating the T1 relaxation of arterial blood and labeling efficiency information enhanced the accuracy of CVR quantification slightly for PCASL and their impacts on VSASL-based CVR measurements were negligible.

### 4.5. Limitations

There are several limitations to this work. One is the need for manual segmentation of the carotid and vertebral arteries from the PC MRI data to estimate the flow volume and velocity. Although the computation was done using automated software, the manual segmentation process might introduce variabilities and partial volume effects to the flow volume and velocity calculation. Thus, a fully automated pipeline to process the PC MRI data is desired to resolve this challenge. Another limitation is the reliance on a single scanner type, which limits the generalizability of the results of studies performed on other scanners, where ASL and PET methods are implemented differently. Among the currently available CVR measurement techniques, phase contrast MRI is often employed as the imaging modality, but it is unable to provide regional CVR quantification.

The voxel-wise analysis of ASL and PET data may be affected by partial volume effects, and yet no consensus was reached on the most effective partial volume correction methods due to the influence of attenuation, estimation of point spread function, and noise models (Aston et al., 2002). This may be worth more systematic analyses in future studies. Although inhaling hypercapnia gas and breath-holding are commonly used stimuli without intravenous injections, we have not sought to examine their impacts on the CVR measurements due to the considerable requirements on gas delivering facilities and the high inter-subject variability of breath-holding duration. A consensus on the optimal imaging technology and stimuli may be desired to enable the wider application of CVR experiments. Finally, the restriction of the study to healthy controls limits our ability to comment on which of the studied ASL methods might perform best in patients with cerebrovascular diseases, as baseline ATT and changes with ACZ will almost certainly differ significantly from the population of healthy subjects.

## Conclusions

In this work, we have investigated the ACZ-induced CVR quantification using simultaneous ^15^O-water PET and ASL MRI. The results from multi-PLD PCASL demonstrated good agreement with the reference PET method. Although the single-PLD PCASL has been the recommended CBF quantification method for clinical imaging, it underestimated CVR because it was unable to account for the significant reduction in ATT caused by the administration of ACZ. The VSASL was a feasible approach in measuring CVR for GM regions but it was limited by the signal contamination near the ventricles. Therefore, we conclude that multi-PLD PCASL is the most desirable ASL technique for CVR measurement in healthy subjects.

## Supplementary Material

1

## Figures and Tables

**Table 1:** PET/MRI scanning parameters.

Parameter	Unit	Value
**PET**		
Matrix		192 × 192
FOV	mm	300
Voxel size	mm	1.56 × 1.56 × 2.78
Slice thickness	mm	2.78
Average Scan duration	min	15:00
**MRA**		
TR/TE	ms	22/2.4
Number of slices		120
Flip angle	degrees	15
Slice thickness	mm	1.2
Matrix		512 × 512
FOV	mm^2^	220 × 220
Voxel size	mm^2^	0.43 × 0.43
Scan duration	min	4:03
**GRE**		
TR/TE	ms	667/15
Number of slices		30
Flip angle	degrees	20
FOV	mm^2^	24 × 24
Matrix		256 × 256
Slice thickness	mm	5
Scan duration	min	1:56
**PC MRI**		
TR/TE	ms	12.4/4.6
Flip angle	degrees	20
FOV	mm	180 × 80
Matrix		512 × 512
Voxel size	mm	0.3516 × 0.3516 × 3
Cardiac phases		10
Time per cardiac phase	ms	88
Slice thickness	mm	3
Number of slices		1
Velocity encoding	cm/s	100
Repeats		2
Scan duration	min	1:30
**Single-PLD PCASL**		
Labeling pulse shape		Hanning
Labeling pulse duration	ms	0.5
Labeling pulse spacing	ms	1.22
Mean B1	*μ*T	1.4
Mean gradient strength	mT/m	0.7
Maximal gradient strength	mT/m	7
Bolus duration	ms	1450
TR/TE	ms	4854/10.7
PLD	ms	2025
NEX		3
Acquisition Matrix		8 interleaved spirals × 512 sampling points per spiral
Number of slices		36
FOV	cm^3^	24
Acquisition Voxelsize	mm	3.73 × 3.73 × 4
Reconstruction Voxelsize	mm	1.875 × 1.875 × 4
Number of Background suppression pulses		5
Scan duration	min	4:13
**Multi-PLD PCASL**		
Labeling pulse shape		Hanning
Labeling pulse duration	ms	0.5
Labeling pulse spacing	ms	1.22
Mean B1	*μ*T	1.8
Mean gradient strength	mT/m	0.7
Maximal gradient strength	mT/m	4.5
Bolus duration	ms	1700
TR/TE	ms	5652/10.7
PLD	ms	300, 2000, 3700
Acquisition Matrix		4 interleaved spirals × 512 sampling points per spiral
NEX		2
Number of slices		36
FOV	cm^3^	24
Acquisition Voxelsize	mm	5.77 × 5.77 × 4
Reconstruction Voxelsize	mm	1.875 × 1.875 × 4
Number of Background suppression pulses		5
Scan duration	min	4:47
**VSASL**		
Bolus duration	ms	1590
TR/TE	ms	4064/10.7
Inversion time (TI)	ms	1600
Pre-delay	ms	1900
NEX		6
Background suppression pulses		3
Acquisition Matrix		4 spirals × 512 sampling points
Number of slices		36
FOV	cm^3^	24
Acquisition Voxel size	mm	5.77 × 5.77 × 4
Reconstruction Voxelsize	mm	1.875 × 1.875 × 4
Vc	cm/s	2
Scan duration	Min	3:26

**Table 2:** Summary of subject information and experimental parameters.

Parameter	Unit	Value Range	Mean	SD
Number of subjects		19		
Male to female ratio		8: 11		
Age	years	25 – 66	37	12
Hemoglobin	g/dL	12.3 – 18.2	14.7	1.91
Hematocrit	%	36 – 53	43	5
Blood oxygen saturation level	%	98 – 100	99.9	0.4
Estimated T1 relaxation of arterial blood	s	1.64 – 1.85	1.75	0.07
^15^O-water administered	MBq	592 – 1022	862	123
Acetazolamide administered	g	0.7 – 1.0	0.96	0.09

## Abbreviations:

ACZ: Acetazolamide
AIF: Arterial input function
ASL: Arterial spin labeling
ATT: Arterial transit time
BRAVO: Brain Volume Imaging
CBF: Cerebral blood flow
CVR: Cerebrovascular reactivity
FOV: Field of view
FWHM: Full width at half maximum
GM: Gray Matter
GRE: Gradient echo
MRA: Magnetic resonance angiography
NEX: Number of excitations
OSEM: Ordered subset expectation maximization
PC: Phase contrast
PCASL: Pseudo-continuous arterial spin labeling
PLD: Post-labeling delay
RMSE: Root-mean-square error
ROI: Region of interest
TCD: Transcranial Doppler
TOF: Time-of-flight
TR: Repetition time
WM: White matter
Vc: cut-off velocity
VSASL: Velocity selective arterial spin labeling
