# Supplementary material for: Cerebrovascular reactivity measurements using simultaneous 15O-water PET and ASL MRI: Impacts of arterial transit time, labeling efficiency, and hematocrit
Source: Neuroimage. Author manuscript; Available in PMC 2021 Jul 10. (PMC8272558; doi:10.1016/j.neuroimage.2021.117955)
Supplement: 1 [file NIHMS1714818-supplement-1.docx]

**Supplementary Materials**

***T1 relaxation of arterial blood***

The T1 relaxation of the arterial blood of each subject was quantified using the following equation:

$$T_{1b}=1/{\{f_{e}\cdot[}1.099-0.057\cdot B_{0}+0.033\cdot Hb\cdot(1-Y)]+[(1-f_{e})\cdot0.496-0.023\cdot B_{0}]\}$$

$$f_{e}=0.70\cdot Hct/[0.70\cdot Hct+0.95\cdot(1-Hct)]$$

where $B_{0}$ is 3.0T, Hb and Hct are the hemoglobin level hematocrit measured in the blood test respectively, and Y is the percentage of oxygen saturation in the arterial blood [1]

***CBF Quantification Using VSASL***

The CBF can be computed using the following model:

$$\Delta M=M_{0}\cdot\alpha\cdot\tau\cdot CBF\cdot e^{-\frac{TI}{T_{1}}}\cdot e^{-\frac{TE}{T_{2}}}$$

where $\Delta M$ is the ASL label and control difference signal, $M_{0}$ is the proton density, $\alpha$ is the labeling efficiency, $\tau$ is the labeling duration, $TI$ is the inversion time [2].

***Discussion on velocity profiles and diffusion attenuation effects of VSASL***

The velocity selective profile of FT-VSI pulse as compared to VSS pulse can be found in Figure 2A in [3], where the velocity cutoff sharpness is almost the same if the FT-VSI pulse is carefully designed, e.g., with the sinc-modulated VSI pulse, whereas the original FT-VSI (rect-VSI) pulse in fact has a less sharp cutoff profile. In addition, due to the repeated “inversion dips” on the magnetization of moving spins at higher velocities, it is not clear whether VSI labeling would have less sensitivity to velocity changes seen in this study.

For the diffusion attenuation effects in the VSS labeling pulse (symmetric BIR8 VSS pulse, symBIR8), it may only contribute for up to 0.033% of the fully relaxed CSF signal, where a b-value of 0.37 s/mm^2^ for the sym-BIR8 pulse and the diffusion coefficient of 0.00089 mm^2^/s for grey matter are assumed.

For FT-VSI pulse, the diffusion compensation is only valid when compensating gradient pulses are applied [4] (Qin and van Zijl, MRM, 2016). However, even with a long pulse duration of 64 ms, the eddy current effects may contribute significant errors to the ASL signal, e.g., up to 1-2% or 4-5% of the tissue signal at 10 cm or 20 cm from the iso-center, respectively (Figure 3C in Qin, et al, MRM, 2016). Though the newly developed sinc-VSI pulse has similarly low eddy current sensitivity, it was not available at the time of the study, therefore the symBIR8 pulse was chosen for its robustness against B1/B0 variation and eddy current effects [3].

Table S1: Slopes of the fitted regression lines between Dynamite PET and ASL techniques in Experiment 1

|  | Dynamic PET vs  Single-PLD PCASL | Dynamic PET vs  Multi-PLD PCASL | Dynamic PET vs  VSASL |
| --- | --- | --- | --- |
| Full Brain | 0.5655 | 0.8010 | 0.6278 |
| Gray Matter | 0.5853 | 0.8030 | 0.5872 |
| White Matter | 0.5327 | 0.7623 | 0.6800 |

Table S2: Slopes of the fitted regression lines between Dynamite PET and ASL techniques in Experiment 2

|  | Dynamic PET vs  Single-PLD PCASL | Dynamic PET vs  Multi-PLD PCASL |
| --- | --- | --- |
| Full Brain | 0.5424 | 0.7715 |
| Gray Matter | 0.5589 | 0.7805 |
| White Matter | 0.5128 | 0.7232 |

Table S3: Parameters for simulated 3-PLD and 7-PLD PCASL data.

|  | 3-PLD PCASL | 7-PLD PCASL |
| --- | --- | --- |
| CBF (ml/100g/min) | 60 | 60 |
| ATT (s) | 1.3 | 1.3 |
| Labeling duration (s) | 1.7 | 1.7 |
| PLD (s) | 0.3, 2, 3.7 | 0.3, 1, 1.7, 2.4, 3.1, 3.8, 4.5 |
| T1 of blood (s) | 1.65 | 1.65 |
| NEX (number of repeats) | 2 | 1 |
| T1 of tissue (s) | 1.3 | 1.3 |
| SNR | 5 | 5 |
| Number of realizations | 1000 | 1000 |


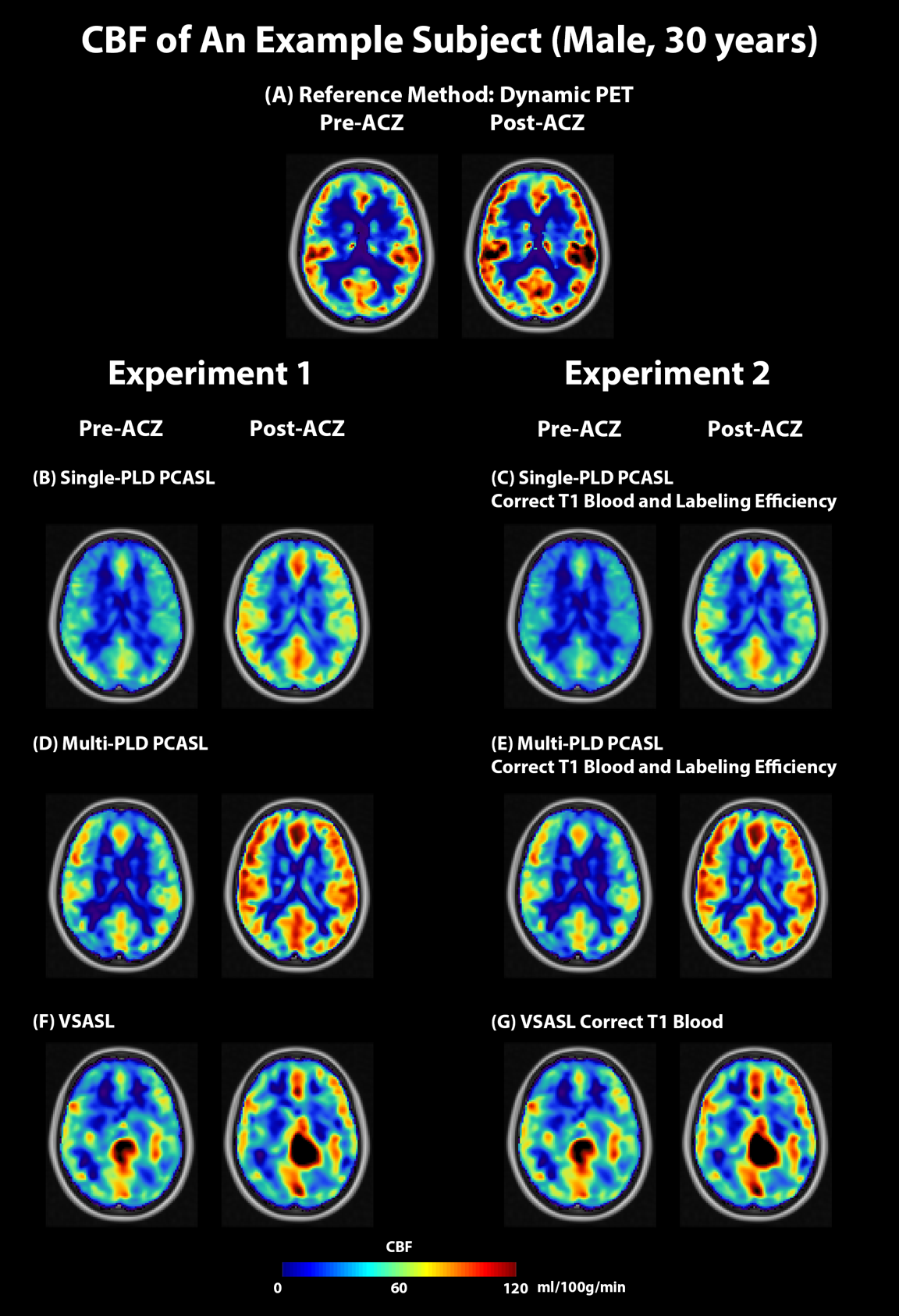


Figure S1: CBF map of an example subject. All imaging techniques detected the increase in CBF induced by ACZ.


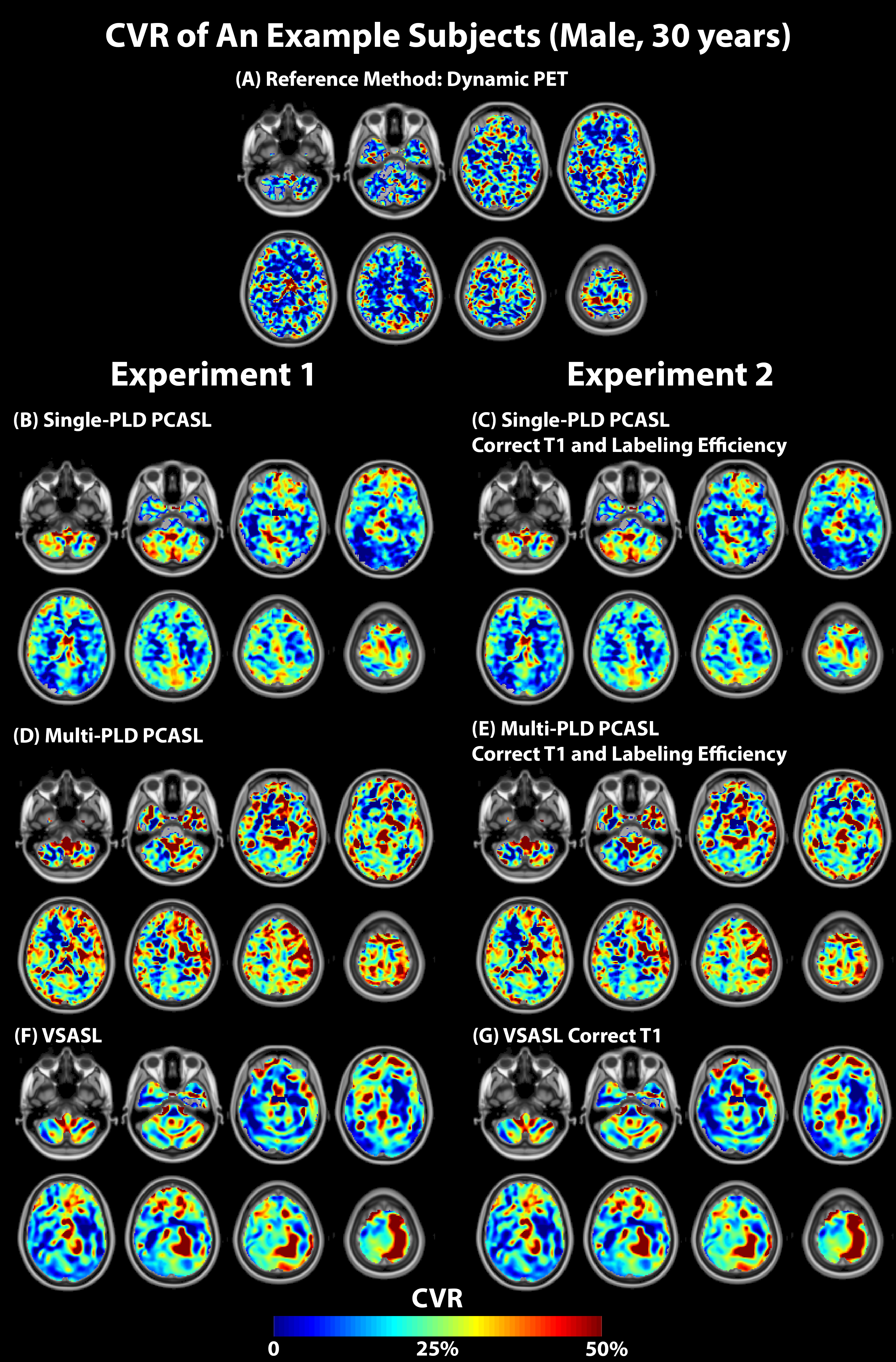


Figure S2: CVR map of an example subject. Using the single-PLD PCASL technique, CVR in the occipital lobe was lower than the values obtained using PET.


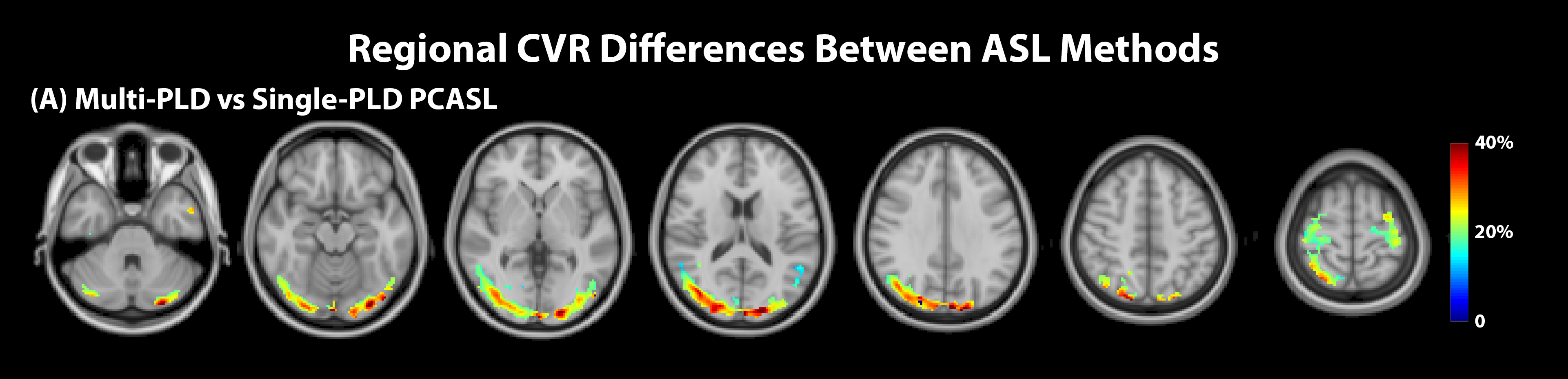


Figure S3: Regions of significant CVR differences (t test; corrected p-value <0.05) between multiple and single PLD PCASL and effect size. The highlighted regions with significant CVR difference clustered in the temporal lobe, where the blood is supplied by the calcarine branch of the posterior cerebral artery with the longest ATT. This implied that regions with single-PLD PCASL underestimated CVR in areas with long ATT.


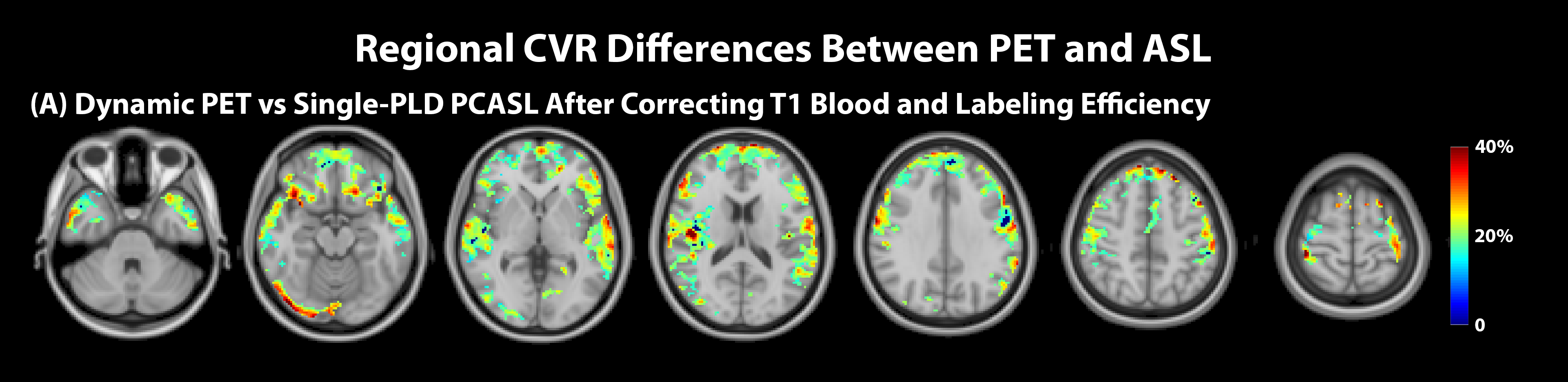


Figure S4: Regions of significant CVR differences (t test; corrected p-value <0.05) between PET and single-PLD PCASL after correction and effect size.


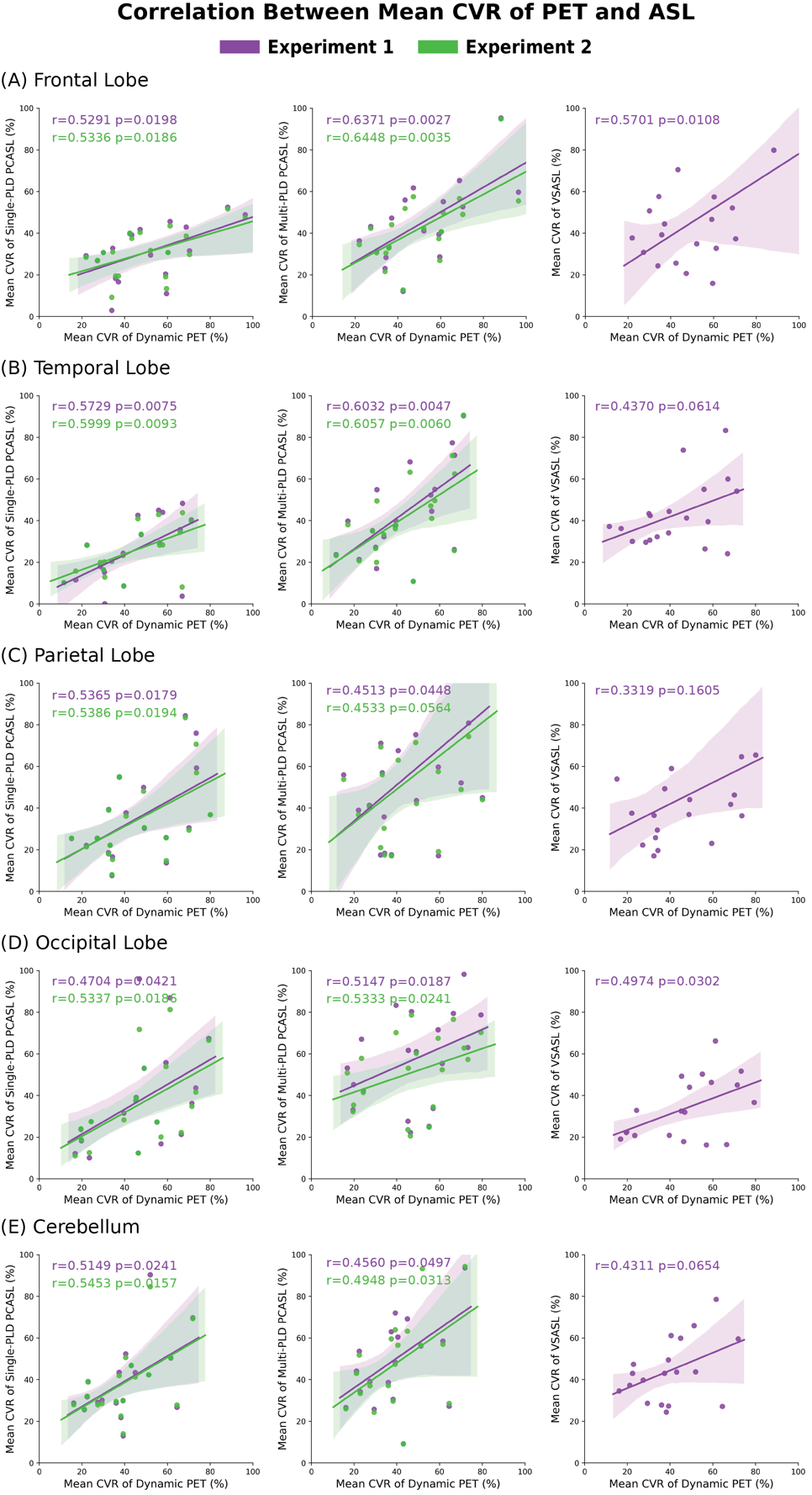


Figure S5: Correlation between the mean CVR measured by PET and ASL methods for different ROIs in both experiments. The shaded area represents the 95% confidence interval.


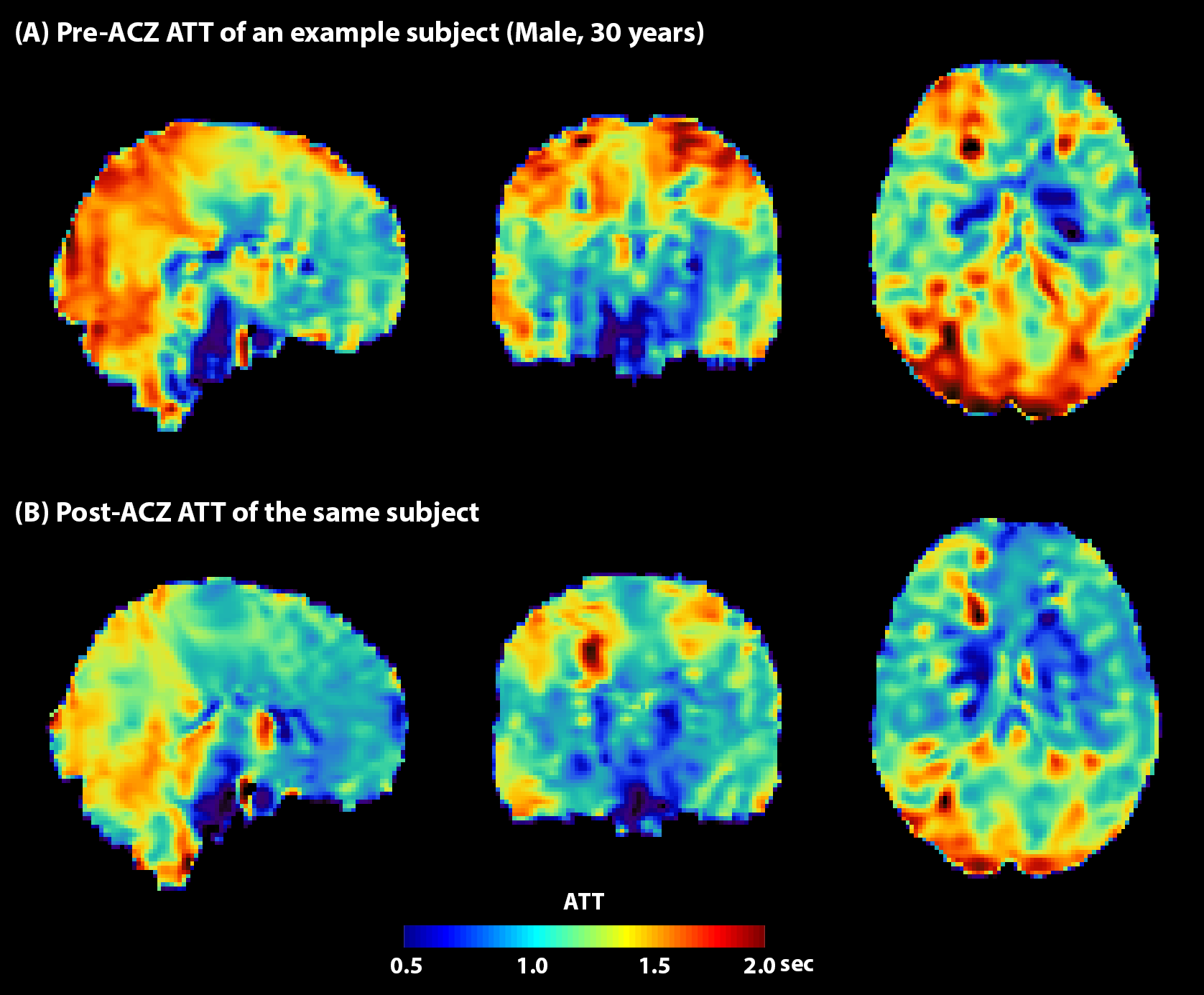


Figure S6: ATT map of an example subject before and after the administration of ACZ.


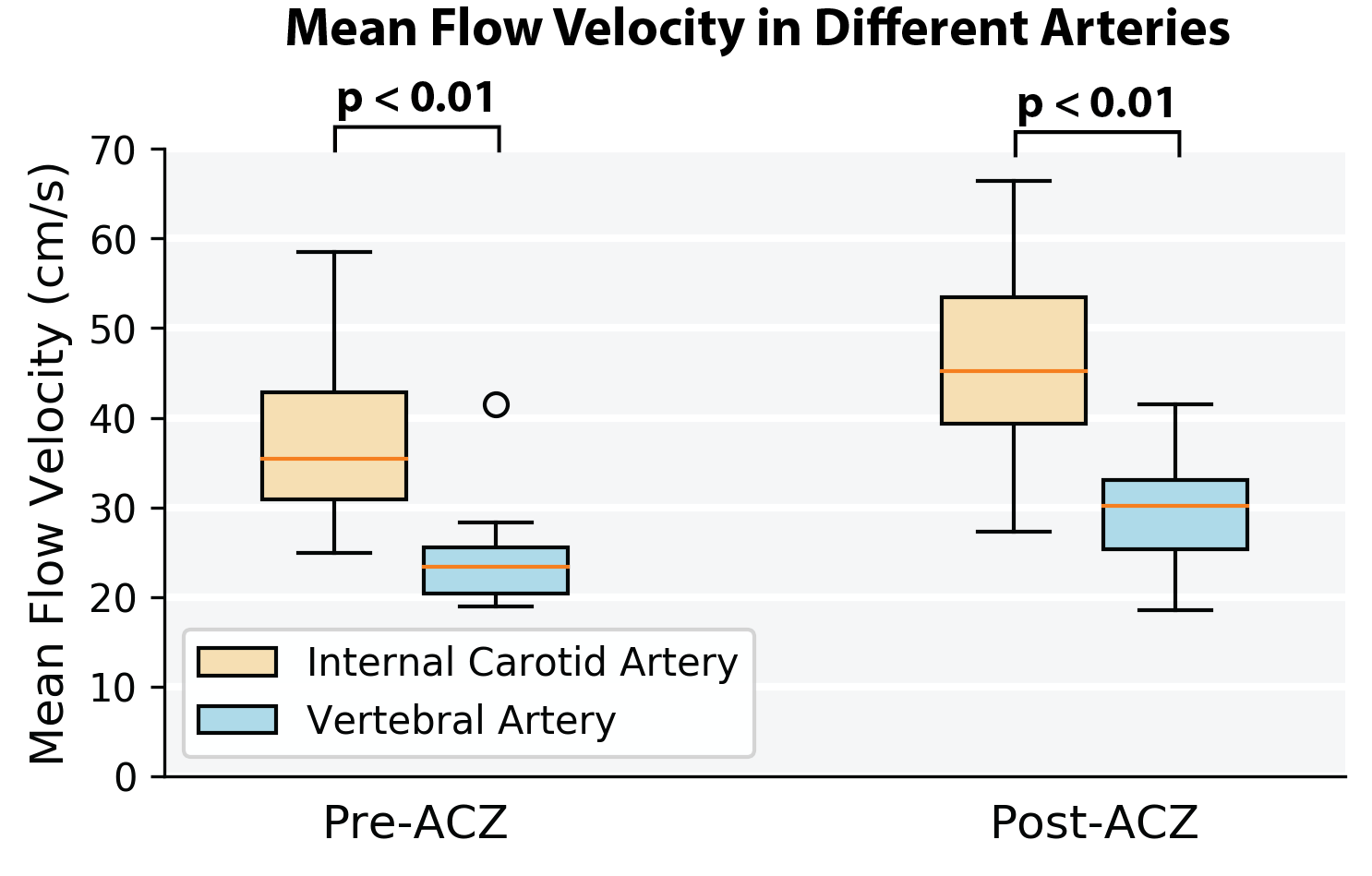


Figure S7: Mean Flow Velocity in the internal carotid artery and vertebral artery. The velocity in the internal carotid artery was significantly higher than in the vertebral artery before and after the administration of ACZ.


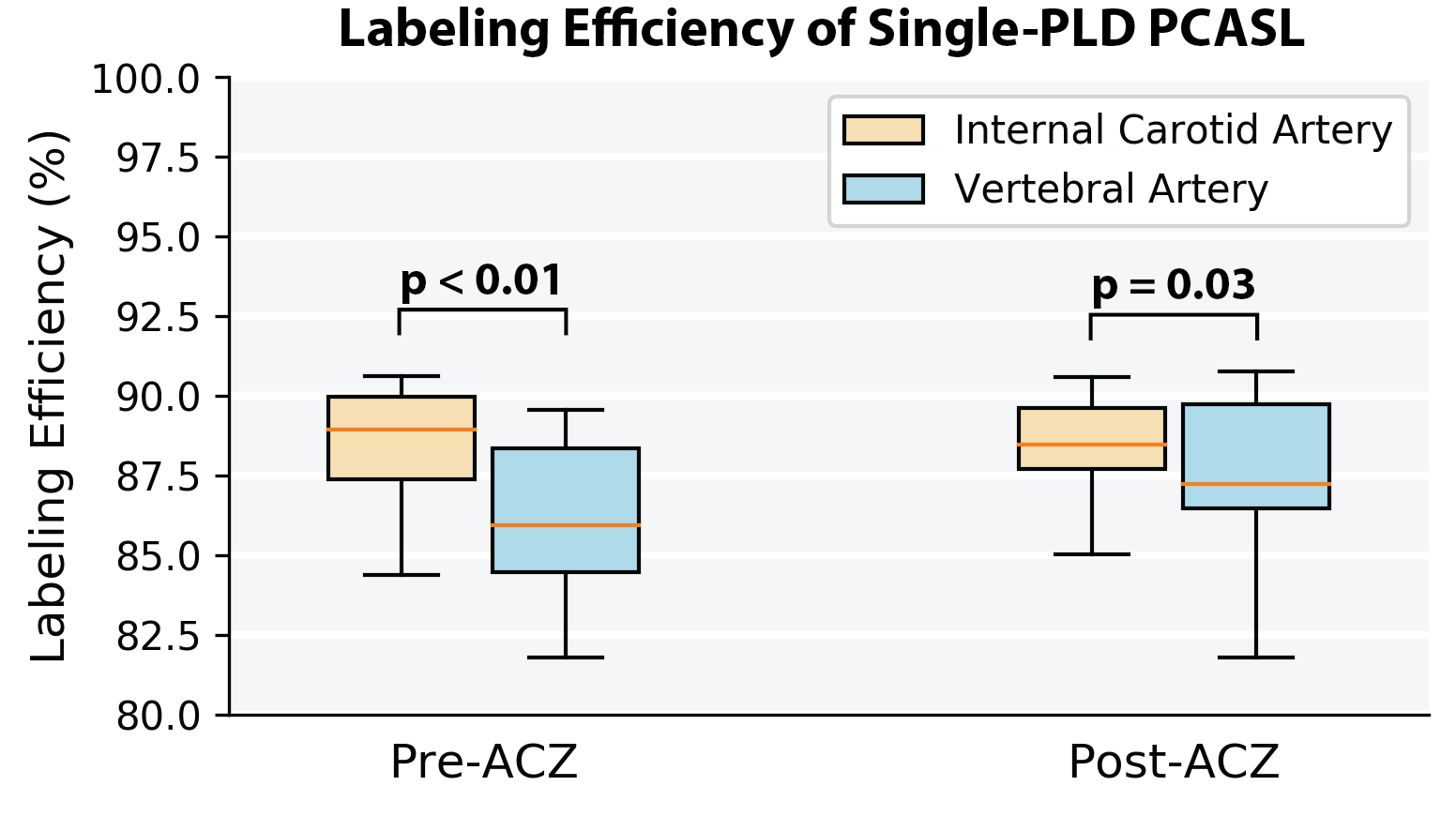


Figure S8: Labeling efficiency of Single-PLD PCASL in the internal carotid artery and vertebral artery. The labeling efficiency in the internal carotid artery was significantly higher than in the vertebral artery before and after the administration of ACZ.


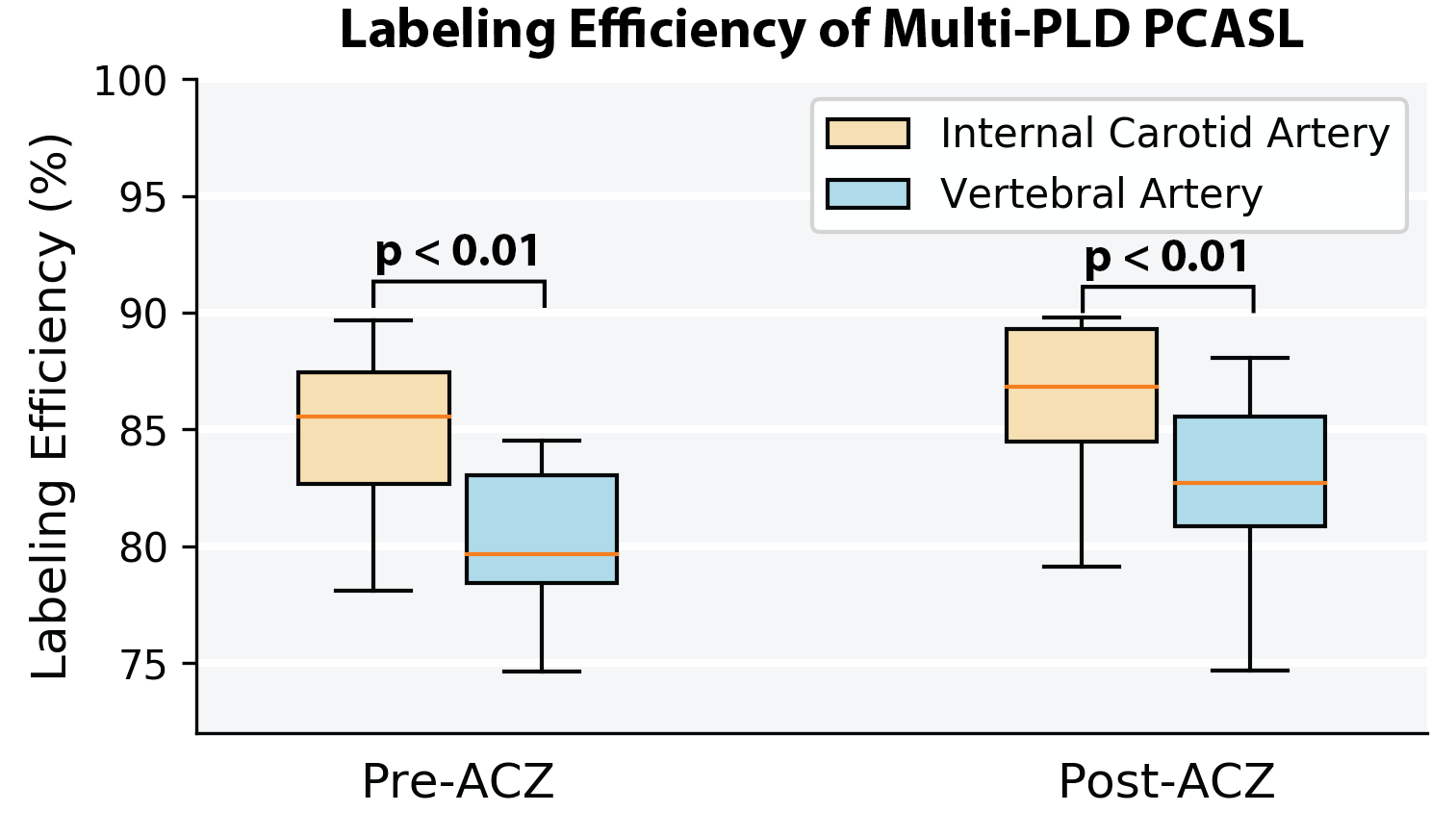


Figure S9: Labeling efficiency of Multi-PLD PCASL in the internal carotid artery and vertebral artery. The labeling efficiency in the internal carotid artery was significantly higher than in the vertebral artery before and after the administration of ACZ.


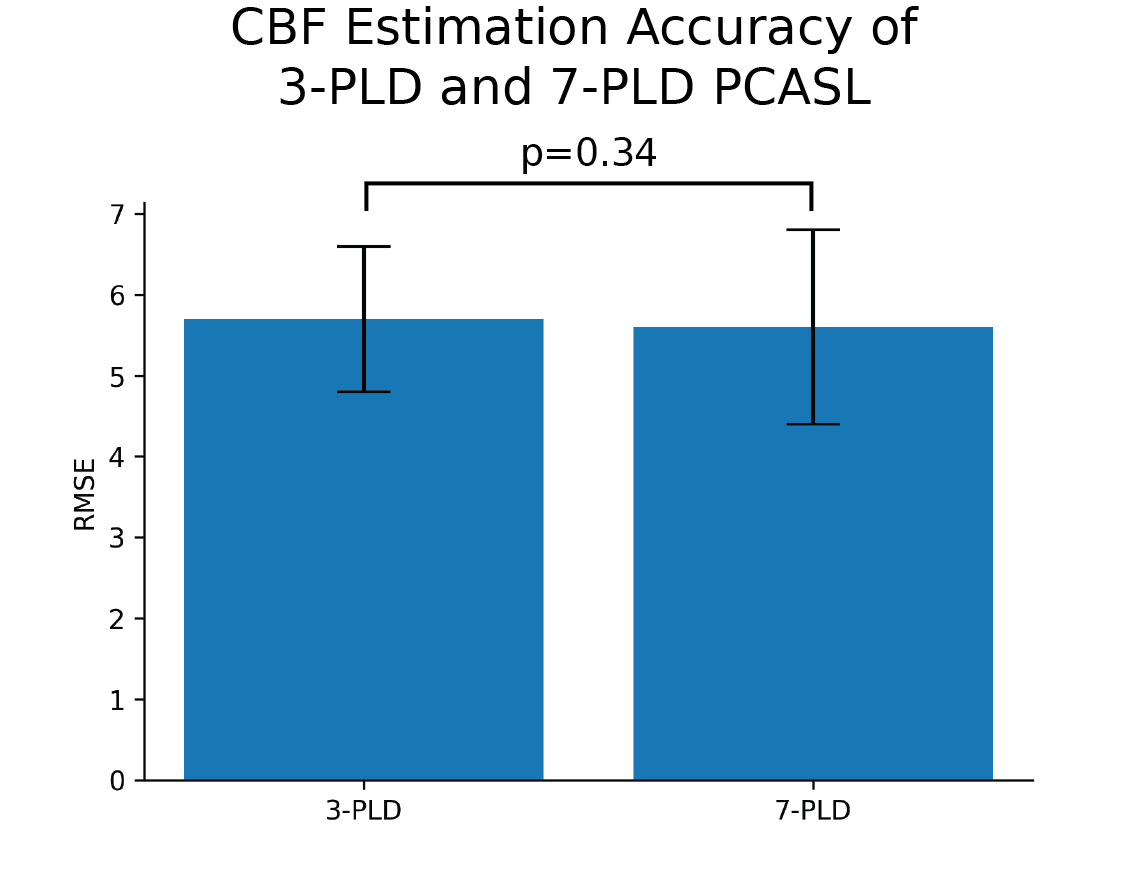


Figure S10: Root-mean-square error (RMSE) between simulated and estimated CBF of 3-PLD and 7-PLD PCASL data (RMSE = 5.7±0.9 and 5.6±1.2 respectively). No significant difference in RMSE was found between these two labeling strategies, indicating that these two labeling strategies were equally effective in CBF quantification for this cohort. The errors of the 3-PLD method were lower due to its higher number of NEX (2 vs 1) for the same acquisition time, making the effective SNR of the 3-PLD technique higher than the 7-PLD method.

References

[1] P. W. Hales, F. J. Kirkham, and C. A. Clark, “A general model to calculate the spin-lattice (T1) relaxation time of blood, accounting for haematocrit, oxygen saturation and magnetic field strength,” *J. Cereb. Blood Flow Metab.*, 2016, doi: 10.1177/0271678X15605856.

[2] E. C. Wong, M. Cronin, W. C. Wu, B. Inglis, L. R. Frank, and T. T. Liu, “Velocity-selective arterial spin labeling,” *Magn. Reson. Med.*, 2006, doi: 10.1002/mrm.20906.

[3] J. Guo, S. Das, and L. Hernandez‐Garcia, “Comparison of velocity-selective arterial spin labeling schemes,” *Magn. Reson. Med.*, vol. n/a, no. n/a, doi: https://doi.org/10.1002/mrm.28572.

[4] Q. Qin and P. C. M. van Zijl, “Velocity-selective-inversion prepared arterial spin labeling,” *Magn. Reson. Med.*, vol. 76, no. 4, pp. 1136–1148, 2016, doi: 10.1002/mrm.26010.
